# Potential of natural products and gut microbiome in tumor immunotherapy

**DOI:** 10.1186/s13020-024-01032-7

**Published:** 2024-11-20

**Authors:** Luchang Cao, Xinmiao Wang, Xinyi Ma, Manman Xu, Jie Li

**Affiliations:** Department of Oncology, Guang’anmen Hospital, China Academy of Chinese Medical Sciences, No.5, Beixian’ge Street, Xicheng District, Beijing, China

**Keywords:** Cancer, Immunotherapy, Resistance, Gut microbiota, Nature product

## Abstract

Immunotherapy is a novel treatment approach for malignant tumors, which has opened a new journey of anti-tumor therapy. Although some patients will show a positive response to immunotherapy, unfortunately, most patients and cancer types do not achieve an ideal response to immunotherapy. Therefore, it is urgent to search for the pathogenesis of sensitized immunotherapy. This review indicates that *Fusobacterium nucleatum*, *Coprobacillus cateniformis*, *Akkermansia muciniphila*, *Bifidobacterium*, among others, as well as intestinal microbial metabolites are closely associated with resistance to anti-tumor immunotherapy. While natural products of *pectin*, *inulin*, *jujube*, *anthocyanins*, *ginseng polysaccharides*, *diosgenin*, *camu-camu*, and *Inonotus hispidus (Bull).Fr. P. Karst*, *Icariside I*, *Safflower yellow*, *Ganoderma lucidum*, and *Ginsenoside Rk3*, and other Chinese native medicinal compound prescriptions to boost their efficacy of anti-tumor immunotherapy through the regulation of microbiota and microbiota metabolites. However, current research mainly focuses on intestinal, liver, and lung cancer. In the future, natural products could be a viable option for treating malignant tumors, such as pancreatic, esophageal, and gastric malignancies, via sensitizing immunotherapy. Besides, the application characteristics of different types, sources and efficacy of natural products in different immune resistance scenarios also need to be further clarified through the development of future immunotherapy-related studies.

## Introduction

In the 1890s, American surgeon William Coley used Coley toxin to treat tumors, pioneering tumor immunotherapy. Following the use of chemotherapy, targeted therapy, and anti-angiogenesis therapy, the introduction of immunotherapy in tumor therapy is a noteworthy development. The efficacy and durability of this novel family of immune checkpoint inhibitors was impressively demonstrated. It consists of cytotoxic T-lymphocyte antigen-4 (CTLA-4) [1], programmed death ligand 1 (PD-L1), and programmed death receptor 1 inhibitors (PD-1) [2]. An increasingly significant advancement with the management for cancer is the inclusion of solid tumors in immune checkpoint inhibitor regimens [3–6]. Immunotherapy for cancer has helped a great deal of patients, however not everyone responds the same to immune checkpoint inhibitor (ICI) treatment. Presently, antibodies against PD-1 and PD-L1 have been approved for treatment in a number of different cancer types [7–9]; however, the objective response rates to these inhibitors vary according to the type of tumor, ranging from 15 to 87% [10, 11]. Even while ICI-based immunotherapy has shown to be highly effective in improving patient outcomes for a variety of cancer types, a sizable portion of individuals fail to achieve a long-lasting response. Specifically, even among patients who respond to therapy with anti-PD-1 the best, 60–70% of patients with melanoma are unable to objectively respond to treatment. Eventually [12, 13], 20–30% of patients with tumors will experience recurrence or progression. To provide immunotherapy's benefits to a wider range of patients, it is imperative that methods for boosting immunotherapy's response rate and conquering immunological resistance be looked into immediately.

The gut is home to a variety of microbes that can cause illness and impact health. They change several physiological systems, such as immune function, metabolism, and digesting capacity, which has a major effect on human wellness and illness [14–18]. The importance of gut microorganisms in controlling tumor immunity has been demonstrated. They can fight immune resistance and non-response by influencing the efficacy of cancer immunotherapy and modulating anti-tumor immune responses. Consequently, regulating intestinal microorganisms offers new insights for improving the effectiveness of tumor immunotherapy and overcoming immune resistance [19–23].

Natural products are naturally occurring small molecules that have shown great potential in treating a variety of diseases [24–26]. In addition to being the main target of drug development, screening, and research, they are also essential for controlling the immune response. Many natural products have recently been discovered to have potent anti-cancer qualities. They can regulate the immune response to tumors by influencing intestinal microorganisms and enhancing the efficacy of tumor immunotherapy. Many compounds that are derived from natural products or their derivatives are either being evaluated in clinical studies or are being used as cancer therapies. This review aims to explain what links intestinal microbiota and the immune response to malignancies, as well as natural chemicals that alter intestinal microorganisms to improve the immune response to immunotherapy. This will further the development of tumor immunotherapy and enhance clinical intervention strategies for immune resistance.

## The connection among tumor immune resistance and gut microbiota

**Fig. 1:**
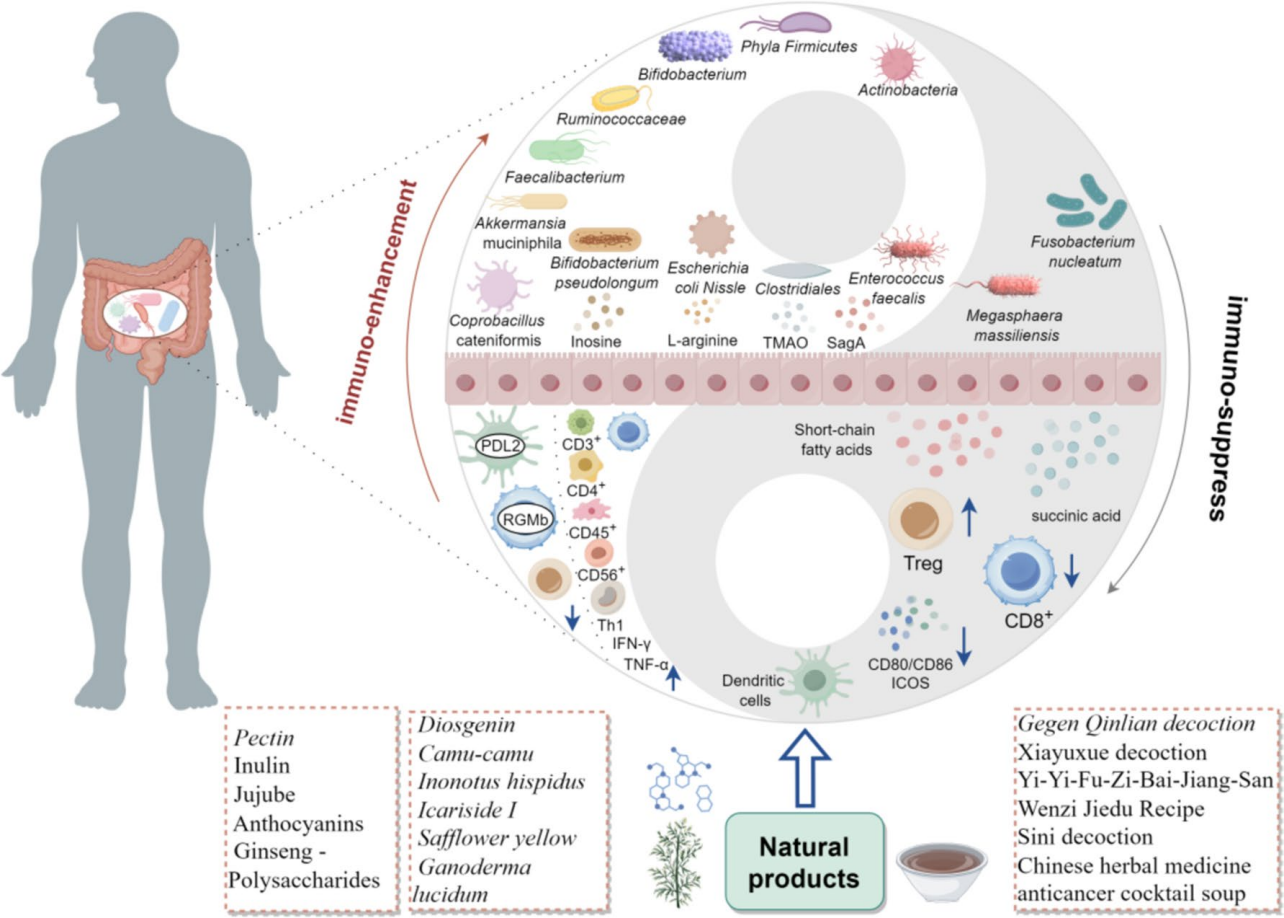
The relationship and mechanism between gut microbiome and tumor immune resistance

The variety of microbes and their metabolites that live in the intestines are known to as "gut microbiota". These microbes influence the body's medicine and diet, among other things. Additionally, they contribute to the modification of immunological state [14, 16, 17]. There are currently research that support the link among gut flora and anti-tumor immune therapy efficacy [19–21]. For example, a group at Peking University Cancer Hospital discovered that the ratio of Prevosia/Bacteroides in patients positively correlated with the effectiveness in anti-PD-1/PD-L1 immune therapy. They also discovered that the abundance of *Lachnospiraceae*, *Prevotella*, and *Ruminococcaceae* was significantly higher in some response subgroups. The analysis was done using 16S rRNA sequencing of stool samples [27]. Furthermore, individuals with varying levels of treatment efficiency showed differences in pathways linked to the manufacture of nucleosides and nucleotides, lipids, glucose metabolism, and short chain fatty acids (SCFAs), showing the possible impact to the microbiome of the gut for the therapeutic effect of immunotherapy. Connection among tumor immune resistance and gut microbiota is shown in Fig. 1.

Relentlessly mounting data from experimental and clinical trials demonstrates the beneficial contribution in the intestinal microbiota and its metabolites to immunotherapy response, being a major modulator of immunity against tumors during immunotherapy, mediated by a "gut microbiota-metabolism-immune interaction" pathway [15, 28]. Changing the gut microbiome offers fresh perspectives on enhancing the immune system's capacity to fight cancer. This section centers on the connection between DRTI and gut microbes, which could offer fresh perspectives on immunotherapy resistance.

### Gut microbiota

#### Fusobacterium nucleatum (F.nucleatum)

For CRC, *F. nucleatum* is the most significant pathogen. For the first time, it was discovered in a study that *F. nucleatum* and its metabolite succinic acid exhibited drug resistance when used in CRC immunotherapy [29]. Researchers discovered that patients with advanced colorectal cancer (CRC) who were unresponsive to immunotherapy had higher serum succinic acid levels and more *F. nucleatum* in their feces, both of which were linked to a poor prognosis. *F.nucleatum* inhibits the cGAS-IFN-β pathway, decreases the production of Th1 chemokines CCL5 and CXCL10, and downregulates the chemotactic and activating function of CD8^+^ T cells by secreting the metabolite succinic acid, which stimulates the SUNCR1-HIF-1α-EZH2 axis of the succinic acid receptor in tumor cells. As a result, PD-1 monoclonal antibody's effectiveness in CRC might be diminished. According to in *vivo* research, the antibiotic metronidazole may reduce a certain amount about *F. nucleatum* at the colon and the serum level of succinic acid, thereby re-sensitizing CRC to immunotherapy.

#### Coprobacillus cateniformis

While PD-1 is a common docking partner for both PD-L1 and PD-L2, PD-L2 can additionally connect to RGMb, which is a binding receptor for PD-L2. The findings showed that resistance to PD-1 or PD-L1 inhibitors caused via the microbiota can be overcome by inhibiting the PD-L2-RGMb interaction [30]. When mice were reconstituted with healthy human microbiota (HMB) instead of germ-free and antibiotic-treated mice injected with MC38 colon cancer, the efficacy for anti-PD-1 /PD-L1 immunotherapy against tumors was increased. By decreasing the expression of RGMb in CD8^+^ T cells and PD-L2 in dendritic cells, microbiota can strengthen immunity against tumors. Conversely, the reduction of PD-L2 expression is required for the symbiotic gut microbiota *Coprobacillus cateniformis* to have an effect on tumor fighting immunity. Anti-tumor immunity can be strengthened when anti-RGMB and anti-PD-L1 therapy are combined.

#### Akkermansia muciniphila

Antibiotic therapy (ATB) is a major contributor to gut microbiota imbalance and has the potential to reduce PD-1's therapeutic efficacy and develop PD-1 resistance. It was discovered that individuals who progression-free survival (PFS) longer than six months have greater metagenomic species and gut microbial gene abundance [31]. The majority of *kkermansia muciniphila* (*A. muciniphila*) is found in patients with stable disease (SD) and partial response (PR) who are receiving effective PD-1 medication therapy. *Firmicutes* is most abundant in long-lived patients. Additionally, *Enterococcus hirae* (*E.hirae*) was most frequently discovered in the stool of PR patients, supporting the association with intestinal microbiota and the effectiveness with immune therapy. *A. muciniphila* bacteria were also found in 69% of PR patients and 58% of SD stool samples. *A. muciniphila* or *A. muciniphila* & *E.hirae* administered intragastrically to mice reversed their PD-1 response to antibiotic therapy. Another study [32] conducted elsewhere discovered that patients via metastatic renal cell carcinoma (RCC) had higher relative abundances and prevalences of *A. muciniphila* and *B. salyersiae* in their stool among ICB responders (Rs) compared to non-responders (NRs). NR-fecal microbiota oral transplantation (FMT) (without *A. muciniphil* or *B.salyersiae*) with the immunostimulant *A. mucinipila* or *B. salyersiae* or R-FMT can restore sensitivity to immunotherapy prior to every ICB period. Because the gut microbiota regulates immunity against tumors in cancer patients, it may be possible to eradicate or reverse patients' immunotherapy resistance by modifying the gut ecosystem.

#### Bifidobacterium

Researchers led by Professor Thomas F. Gajewski of the University of Chicago examined certain bacteria in 42 patients about advanced melanoma both before and after receiving anti-PD-1 therapy. They found that the amounts of *Bifidobacterium longum*, *Collinsella aero faciens*, and *Enterococcus faecium* were higher in individuals who reacted favorably to immunotherapy [33]. Among them, *Bifidobacterium* OTU (559527) had the highest Spearman correlation with *Bifidobacterium* longum, and mice receiving these bacteria responded more strongly to T cells and immunotherapy. Moreover [34], *Bifidobacterium* was linked favorably to anti-tumor T cell responses. Mice given with *Bifidobacterium* had considerably better tumor control than mice not treated with *Bifidobacterium*, and this improvement was supported by an effective induction of peripheral tumor-specific T cells and an increase in the buildup of CD8^+^ T cells specific to the antigen within tumors. As a result, oral *Bifidobacterium* treatment could enhance the effectiveness of ICIs, increase anti-tumor immunity, and maybe strengthen the therapeutic effect of immune checkpoint inhibitors such as CD47, PD-1/PDL-1, and CTLA4 inhibitors.

#### Ruminococcaceae

Researchers led by Professor J.A. Wilgo of the MD Anderson Cancer Center have discovered [35] that Rs to melanoma PD-1 immunotherapy possess substantially greater alpha diversity and proportion of the *Ruminococcaceae* family, and that R has more functional anabolic pathways of gut bacteria than NRs. There is also increased anti-tumor and systemic immunity. Individuals possessing a "good" gut microbiome, defined as having a high variety and abundance of *Rumenococcaceae/Faecalis*, were found to mediate a strengthened systemically and anti-tumor immune system response. This was achieved by improved activity of effector T cells in the tumor microenvironment and higher antigen presentation. Due to restricted lymphocyte and myeloid infiltration, decreased antigen-presenting potential within the tumor, and poor variety and high relative abundance of *Bacteroides* in their gut microbiome, those who had "unfavorable" gut microbiomes mediated a compromised systemic and anti-tumor immune response. Furthermore, it was discovered that patients with high gut microbial abundances of *Clostridiales*, *Ruminococcaceae*, or *Faecalibacterium* showed increased systemic circulation levels of effector CD4^+^ and CD8^+^ T cells together with an anti-PD-1 therapy response. Conversely, patients whose gut microbiome included a high concentration of *Bacteroidales* had higher amounts of regulatory T cells (Treg) and myeloid-derived suppressor cells (MDSC) in the bloodstream, along with a lower cytokine response.

#### Faecalibacterium

Using the method of 16S rRNA gene sequencing, a study assessed the diversity of the microbes in the feces at baseline and prior to each treatment in patients of metastatic melanoma (MM) receiving ipilimumab [36], an immune checkpoint inhibitor that targets CTLA-4. Contrary to cluster B patients, that baseline microbiota had been altered primarily Bacteroides, it was discovered that the baseline microbiota for cluster A was enriched for *Faecalibacterium* along with other *Firmicutes* as unclassified *Ruminococcaceae*, and *Clostridium* XIVa. *Blautia* and *Clostridium* XIVa exhibited prolonged OS and PFS. Cluster A and long-term clinical benefit were linked to a low number of peripheral blood tregs and a considerably reduced percentage of baseline α4β7 CD4^+^ and α4β7 CD8^+^ T cells. It is clear that the clinical advantage of increased efficacy to immunotherapy targeting CTLA-4 is connected with a baseline gut microbiota richer in *Faecalibacterium* and other *Firmicutes*. There is more *Bacteroidetes*, especially the *Bacteroidetes* genus, among individuals that fail to respond well with immunological treatment.

#### Phyla Firmicutes and Actinobacteria

In the single-arm clinical trial NCT03341143 [37], it was discovered that following a single FMT in PD-1 inhibitor Rs patients and non-responders (NRs), the flora of complete response (CR) donors in PD-1 inhibitor Rs patients showed higher α-diversity. Individuals with PD-1 refractory melanoma are those who have not responded to PD-1 inhibitors in the past, either on their own or in combination with CTLA-4 or the experimental medication. While the majority of the groups with a substantial drop in Rs belonged to the phylum *Bacteroidetes*, the majority of the bacteria that were considerably increased in Rs related to the phyla *Firmicutes* (families *Lachnospiraceae* and *Ruminococcaceae*) and *Actinobacteria* (families *Bifidobacteriaceae* and *Coriobacteriaceae*). In contrast to NRs, Rs exhibited enhanced stimulation of CD8^+^ T and MAIT cells, a higher percentage of CD56^+^CD8^+^ T cells following treatment, and a lower frequency of circulating IL-8 and IL-8-producing myeloid cells. We conclude that FMT combined with PD-1 blockers may change the tumor microenvironment and boost sensitivity to PD-1 inhibitors for the positive effects of immunotherapy in some patients experiencing advanced melanoma.

### Gut microbiota metabolites

There are intricate relationships between gut microorganisms, microbial metabolites, and host cells. The gut microbiome influences several energy metabolism systems in *vivo*. By controlling the immune system, inflammatory response, TME signaling pathway, causing epigenetic alteration, and regulating the inflammatory response, the metabolites generated from gut bacteria play a variety of roles in carcinogenesis and development. They've got an essential contribution to the immune response against tumors.

#### Peptidoglycan hydrolase secretes antigen A

Peptidoglycan hydrolase secretes antigen A (SagA), an enzyme secreted by *Enterococcus* that breaks down bacterial cell wall components and releases fragments of muramyl peptide. These peptide fragments then function as signaling molecules to stimulate the innate immune sensor protein NOD2 and improve the response to immunotherapy. Studies have found [38] that specific *Enterococcus*, mainly *Enterococcus faecium*, *E.durans*, *E.irae* and *E.Meundtii*, have the ability to enhance the potency of anti-PD-L1 immunotherapy for cancer. By comparing the four types of *Enterococcus* that can support immunotherapy and the ineffective *Enterococcus faecalis*, SagA is found in the active *Enterococcus faecium*, and if the *Enterococcus faecalis* has SagA through transgenic technology, they also have the ability to support immunotherapy. If the non-*enterococcus* family of *Lactococcus lactis* gets SagA, it can also become a small anti-cancer helper. Furthermore, following *Enterococcus faecalis* colonization, there was an increase in the overall quantity of CD45^+^ leukocytes and CD3^+^ lymphocytes within the tumor, as well as an increase in the proportion of CD8^+^ T cells infiltrating the tumor. These results imply that anti-tumor cytotoxic T cells boosted innate immunity, created an environment that was favorable for immunotherapy, and improved the effectiveness of immunotherapy.

#### Trimethylamine N-oxide

Initially, it was suggested that trimethylamine N-oxide (TMAO) played a part in preserving the functional and structural strength of proteins. The theory that TMAO can cause inflammation and immunological activation is supported by recent research. Tumors with an active immunological microcosm were shown to have higher levels of the *Clostridiales*-related metabolite TMAO [39]. The combination intramuscular administration of TMAO has a more substantial suppressive impact on growth of tumors than anti-PD-1 antibody alone. It improves immunotherapy response. Through the activation of the endoplasmic reticulum kinase PERK, TMAO causes tumor cells to undergo pyroptosis. In *vivo* triple-negative breast cancer (TNBC) is improved by this procedure in terms of CD8^+^ T cell-mediated immunity against tumors. It stimulates M1 macrophages and CD8^+^ T cell infiltration, which eventually improves CD8^+^ T cell activity, and increases interferon-γ (IFN-γ) and tumor necrosis factor-α (TNF-α) levels. According to a different study [40], TMAO directly boosts effector T cells' ability to fight tumors, boosts macrophage immune response, and lowers the incidence of pancreatic ductal adenocarcinoma (PDAC). Increased survival of patients and an anti-PD-1 response were substantially positively correlated with levels of CUTC^+^ bacteria, a crucial enzyme that catalyzes the synthesis of TMAO. As a result, the gut microbiota's generation of TMAO stimulates an advantageous reaction to immunotherapy.

#### Short-chain fatty acids

Short-chain fatty acids (SCFAs) are vital substances produced by the gut microbiota from fermented fiber in the diet. The amount of SCFAs in the lumen of the intestinal tract increases when complex, indigestible carbohydrates are consumed. *Megasphaera massiliensis*, a low-abundance human gut bacterium, may produce substantial amounts of valerate SCFAs and butyric acid. Elevated levels in the blood of the compound butyrate and propionate have been linked to increased proportion of treg cells and resistance to CTLA-4 inhibition [41]. Butyric acid was discovered to prevent cancer-specific T cells and memory T cells from accumulating in *vivo*, as well as the increased levels of CD80/CD86 on anti-CTLA-4-induced dendritic cells and ICOS in T cells. SCFA may have restricted the anti-CTLA-4 response in patients where moderate iprilizumab with elevated blood butyrate concentrations produced memory, buildup of ICOS^+^CD4^+^ T cells, and infiltration of IL-2. Furthermore [42], valerate or supernatant was used to treat CAR T cells and cytotoxic T lymphocytes (CTLs) that were co-cultured with *Megasphaera massiliensis*. It has the ability to inhibit class I histone deacetylases (HDACs), increase the activity of mTOR, and improve the ability to combat tumors of CTL and CAR-T cells. This is a great illustration of how microbial metabolites might be used therapeutically to improve immunotherapy by affecting both metabolism and epigenetic condition of CTL reprogramming.

#### Inosine

The main source of isosine, a purine metabolite, is the symbiotic bacterium *Bifidobacterium pseudolongum* in the intestinal lumen. It was discovered [42] that inosine can stimulate mucosal Th1 cell proliferation, allowing the cells to move to the tumor microenvironment and strengthen the tumor-opposing immune response. Furthermore, inosine activates A2RR-CAMP-PKA-dependent signaling, that stimulates Th1 proliferation as well as amplifies the immune response against tumors during immune checkpoint blockage (ICB). In addition, by increasing T-cell-mediated cytotoxicity, inosine can produce a "inflammatory" immunological milieu [43]. This has the potential to make ICB effective against cancers such as melanoma and breast cancer, which are usually resistant to it. Additionally, inosine can boost anti-PD-1 and anti-CTLA-4 antibody efficacy.

#### Succinic acid

Succinic acid produced from *F. ucleanum* suppresses the anti-tumor response by preventing CD8^+^ T lymphocytes from entering the tumor microenvironment (TME) in *vivo* and blocking the cGAS interferon-β pathway. Reducing intestinal *F. nucleanum* abundance, which lowers serum succinic acid stages, and raising succinic acid levels in individuals at advanced CRC who do not react to immunotherapy can resensitize tumors through in *vivo* immunotherapy [29].

#### L-arginine

L-arginine has been recognized in the past as a nutrient and has recently been identified as an important immune stimulant. The surface of most tumor cells overexpresses arginine-degrading enzymes, leading to a scarcity of L-arginine in the tumor environment. Apoptosis and T cell malfunction are the outcomes of this paucity [44]. Moreover, L-arginine degradation lowers CD3 expression and decreases T cell responsiveness. Combining treatment with α-PD-L1 antibody and L-arginine was reported to increase the survival rate of patients with osteosarcoma [45]. Additionally, it raises the percentage of CD8^+^ T cells as well as CD86^+^CD11c^+^ dendritic cells within the spleen in *vivo*, upregulates serum IFN-γ levels, and increases the quantity and function of CD8^+^ T cells in tumor-draining lymph nodes. Supplementing with L-arginine boosted the reaction of PD-L1 blocking antibodies and raised the quantity of tumor-infiltrating lymphocytes (TILs). In one study [46], an engineered probiotic bacterium of *Escherichia coli Nissle* 1917 was created using synthetic biology and modified microbial therapeutics. This strain colonizes tumors and produces L-arginine from accumulated waste ammonia. When paired with PD-L1 blocking antibodies, just one boost in L-arginine content within the tumor may increase the amount of T cells invading the tumor and strengthen the immune response.

## Natural products modulate DRTI through microbiota and microbiota metabolites

The clinical response rates to immune checkpoint inhibitors (anti-PD-1/PD-L1) range restricted 10–30% [47], mostly because of the complex tumor micro environment and immune reconstitution mechanisms employed by cancer cells [48]. Modification of the gut microbiota has been frequently applied to control cancer immunotherapy [49].

**Fig. 2:**
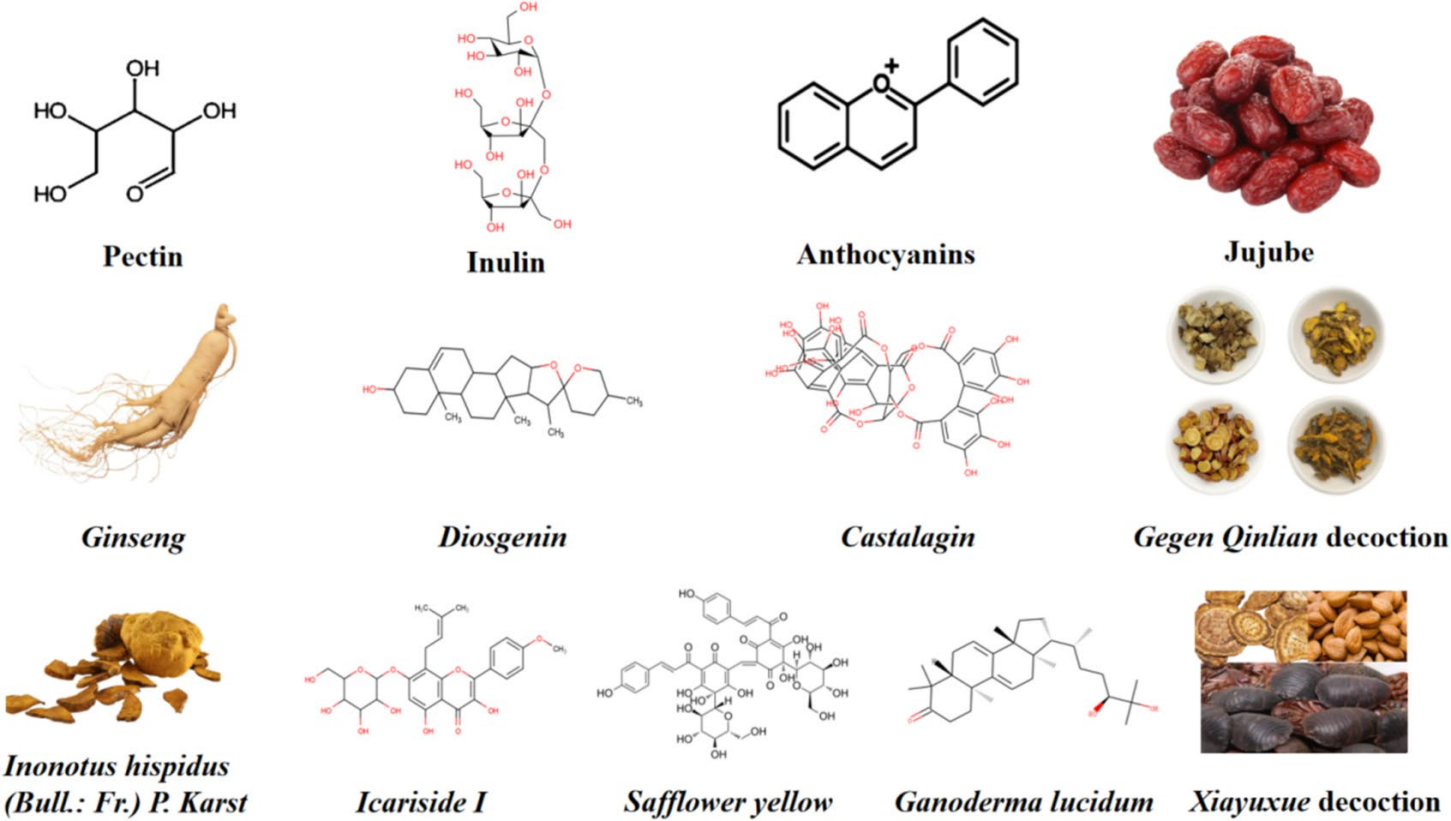
Molecular formula of natural products with DRTI

A growing body of research indicates that a variety of natural compounds, including *curcumin*, *ginseng*, and *berberine*, may improve immune function by changing the tight connection structure, microbial metabolites, and gut microbiota composition. All things considered, elucidating the immunoregulatory impacts of various natural products on gut microbiota may result in novel developments in immunotherapy and cancer treatment. The molecular formula of natural products with DRTI is detailed in Fig. 2.

### Natural active ingredients

#### Pectin

*Pectin* is a commonly ingested soluble fiber that has been shown to reduce gut microbiota imbalances and inhibit CRC by preventing tumor cells from proliferating [50, 51]. Zhang et al. [51] discovered that pectin may improve the anti-PD-1 effectiveness and govern the gut microbiota in mice with humanized microbiomes. Pectin supplementation also significantly increased the levels of several probiotics, including *Erysipelotrichaceae*, *Bifidobacteriaceae*, *Lactobacilllaceae*, and *Ruminococcaceae*, and it can stimulate the infiltration of T cells into the TME and elicit an anti-tumor impact that is dependent on CD8^+^ T cells. Moreover, *Pectin* can help correct an imbalance of the microbes in the gut. The relative number of microorganisms that produce butyrate increased dramatically within the anti-PD-1 combined *Pectin* group, indicating that butyrate plays a positive role in anti-PD-1 monoclonal antibody.

#### Inulin

Dietary fiber known as *inulin*, which is widely distributed in the *Asteraceae* family [52], has been described to be a prebiotic that stimulates the proliferation of *Bifidobacterium* and *Lactobacilli* and strengthens the body's defenses against anti-PD-1 [53, 54]. The proportions of important commensal microorganisms, including *Lactobacillus*, *Roseburia*, and *Akkermansia*—which have been identified as the main producers of SCFAs like acetate, propionate, and butyrate—were elevated by oral delivery of *inulin*-gel therapies [55, 56]. The systemic anti-tumor efficacy of α-PD-1 treatment can be increased by it as it can promote memory recall responses for interferon-γ^+^ CD8^+^ T cells with the development of stem-like T-cell factor-1^+^ PD-1^+^ CD8^+^ T cells within the TME. Furthermore, the concentrations of hippurate, 1-methyl-L-histine, 3-methyl-L-histine, L-arginine, and oleate were significantly elevated by *inulin* gel with α-PD-1 therapy. It's noteworthy to consider that those metabolites have been scientifically linked to a high immunotherapy response rate (AUC = 0.91) [57]. Therefore, systemically memory T cell responses, gut microbiota modulation, and improved responsiveness to checkpoint inhibitors can all be achieved using oral *inulin* gels.

### Jujube

The natural fruit known technically as *Ziziphus jujuba Mill.*, or *jujube* (Chinese date) is frequently utilized in traditional Chinese medicine(TCM). Its antibacterial capabilities, anticancer properties, immunostimulating traits, and antioxidant as well as anti-inflammatory actions are only a few of its many medical benefits [58–62]. According to a study, αPD-L1's increased antitumor efficiency can be attributed to the use of ultrafine *Jujube* powder (JP) [63]. The substantial amount of *Clostridiales*, such as *Lactobacillaceae*, and *Ruminococcaceae*, along with the augmentation of SCFA production—which has a positive correlation to CD8^+^ T-cell infiltration in the tumor—indicate that JP modifies the composition of the gut microbiota. JP of fewer particles can boost the number of CD8^+^ T cells in lymphocytes that infiltrate tumors [64, 65]. As a result, the response of patients to anti-PD-L1 therapy for CRC was significantly enhanced. It demonstrated that ultrafine JP can enhance systemic immunity and tumor immune infiltration by changing the gut microbiota's composition, which enhances αPD-L1's antitumor response.

#### Anthocyanins

*Polyphenolic* chemicals called *Anthocyanins* have been shown to have antioxidant properties and to have anti-tumor properties against colon and breast malignancies [66, 67]. Furthermore, *Anthocyanins* metabolic products generated by gut microbes may be responsible for the anticancer effects [68]. It was established by a study that supplementing with oral *Bilberry anthocyanin* (BA) extracts improves αPD-L1's anti-tumor activity by changing the gut microbiota's makeup [69]. The quantity of *Lactobacillus johnsonii* and *Clostridia* may rise in response to BA extracts. *Clostridia* overexpression would encourage SCFA synthesis and trigger T cell reactions [70, 71]. Furthermore, butyrate concentration and percentage in stools can both be increased by BA, and intratumoral CD8^+^ T cell infiltrations can be strengthened [72]. One of *Lactobacillus Johnsonii's* key functions is immunoregulation. Although oral supplementation of BA extracts with αPD-L1 increased their anti-tumor efficacy, administering BA extracts alone had no discernible effect on the rate at which tumors grow. When combined, BA may affect the gut microbiota's composition, ICIs' efficacy is thereby increased.

#### *Ginseng* polysaccharides

*Ginseng* polysaccharides (GPs), which are extracted from *Panax ginseng*, have been demonstrated to govern the gut microbes, enhance intestinal metabolism, and modify immunomodulatory effects [73]. A combination regimen with GPs and an αPD-1 mAb was found to improve the ratio of CD8^+^/CD4^+^ T cells and downregulate FoxP3 Treg cells in both tumor and peripheral tissues [74]. Additionally, the anti-tumor immunological response is improved by an increase in SCFA, valeric acid abundance, imbalances of indoleamine 2,3-dioxygenase (IDO) activity, and an increase in L-tryptophan production and a decrease in L-kynurenine production in addition to the kynurenine/tryptophan ratio. In addition, there was a notable rise in the number of *Bacteroides*, particularly *B. vulgatus* and *P. distasonis*. These findings suggested that the combo therapy might change the microbiota of the gut towards NRs to Rs in order to increase sensitivity of αPD-1 mAb treatment. Consequently, GPs and αPD-1 mAb could be a novel way to introduce anti-PD-1 immunotherapy to patients with non-small cell lung cancer(NSCLC).

#### Diosgenin

*Diosgenin*, a naturally occurring steroidal saponin, has anticancer and immunomodulatory properties and is derived from the genus Dioscorea [75, 76]. An experiment revealed that the co-administration of *diosgenin* and PD-1 mAb exacerbated tumor necrosis and apoptosis while also improving tumor growth inhibition [77]. The anti-melanoma impact of *diosgenin* appeared to be dependent more on immunity against tumors than on direct tumor suppressive activity, as demonstrated by the tumor tissues' clear production of IFN-γ and CD4^+^/CD8^+^ T-cell infiltration. *Diosgenin* may also improve the composition of the gut microbiota. Following the administration of *diosgenin*, there was a notable increase within the abundance of the *Sutterella* and *Lactobacillus* genera of the *Proteobacteria phylum* and the *Bacteroides* genus from the *Bacteroidetes phylum*, respectively. Nevertheless, CD4^+^/CD8^+^ T- cell infiltration and IFN-γ expression were both reduced when the gut microbiota was disrupted by an antibiotic cocktail technique. This proved that the immune response against tumor induction mediated by *diosgenin* involved the gut microbiota as a mediator.

#### Camu-camu

By controlling the gut microbiota, the Amazonian fruit known as *Camu-camu* (CC) has been demonstrated to have prebiotic benefits that protect mice from obesity and associated metabolic diseases [78]. The primary bioactive ingredient of *CC-Polyphenol* with anticancer action is *polyphenol castalagin*, which is abundant ellagitannin [79]. *Castalagin* affects the composition of the microbiota in a dose-dependent way and avoids αPD-1 resistance in mice given by FMT from NR patients with NSCLC. It was discovered [80] that *castalagin* treatment can reduce *Lachnosclostridium* and enhance the proportions of *Alistipes*, *Ruminococcus*, *Paraprevotella,* and *Christensenellaceae R-7* group. *Castalagin* may stimulate taurine-conjugated bile acids, downregulate secondary taurinated bile acids, and raise the CD8^+^/CD4^+^FOXP3 T-cell ratio when paired with isotype control for αPD-1 (IsoPD-1). It directly engages with *Ruminococcus*'s cellular envelope, promoting an anticancer reaction.

#### *Inonotus hispidus* (Bull.: Fr.) P. Karst

The annual wood rot medicinal fungus *Inonotus hispidus* (Bull.: Fr.) P. Karst., commonly referred to as "*Sanghuang*," is found in the temperate zone and gets its name from parasitic mulberry trees [81]. The spore powder (IHS) of this fungus contains polyphenols and triterpenoids that have a variety of pharmacological effects [82]. According to reports [83], IHS can decrease the abundance of *Allobaculum* and *Mucispirillum* while increasing its prevalence to *Oscillospira*, *Odoribacter*, *Rikenella*, *Dehalobacterium*, and *Coprococcus*. The IHS treatment increased the amount of L-arginine (L-Arg), which is beneficial for CD8^+^ T cells [84]. This suggests that the gut microbiota plays a significant role in the anti-CRC advantages associated with IHS. Additionally, it has the ability to control the immune function mediated by the gut microbiota, namely through increasing the number of CD8^+^ T cells and demonstrating anti-CRC characteristics. Therefore, IHS can regulate the gut microbiome and body immunity, and has anticancer effects, which is a potential drug choice for enhancing anti-tumor immune response by regulating the gut microbiome in the future.

#### Icariside I

Natural plant flavonoids called *Icariside I*, which were isolated from *Epimedium*, have a range of biological actions, including anti-tumor properties [85, 86]. *Icariside I*'s immunological anti-tumor efficacy was further reinforced by a notable increase in a number of lymphocytes, involving CD4^+^ T, CD8^+^ T, NK, and NKT cells, demonstrating an improvement in host immune function. It is commonly known that CD4^+^ and CD8^+^ adaptive T cells, among others, may trigger cytotoxic responses for anti-tumor immunotherapy [87]. Probiotics like *Lactobacillus* and *Bifidobacterium spp.* are significantly increased in response to *Icariside I* treatment, and their ability to promote antitumor immunity and enhance the activity of various lymphocyte subsets has been shown to suppress tumor growth [88]. Additionally, there was an increase in metabolites such as SCFAs and indole derivatives, which have been demonstrated to have an immunoregulation function and which contribute to the regulatory of host metabolism via colonic Gpr41/Gpr43 and AhR signaling [89, 90]. According to this study, *icariside I* regulates the gut microbiota and the metabolites that are produced from it, thereby enhancing the individual's gut barrier and immunity. In order to boost anti-tumor immune responses in the future, more research on this medication option is required.

#### Safflower yellow

*Carthamus tinctorius*, a traditional Chinese medicine, contains *safflower yellow* (SY), the primary active component that has been identified and shown to have anti-tumor biological activity [91]. According to the study, SY and cyclophosphamide (CTX) together shown enhanced therapeutic efficiency in preventing the occurrence of liver tumors and extending the survival of mice with DEN-induced HCC. In the hepatocytes of the DEN-induced HCC mice model [92], SY inhibited the generation of inflammatory factors and reduced liver damage whether taken alone or in combination with CTX. Furthermore, SY can enhance the immunological milieu in DEN-induced HCC by promoting CD8^+^ T cell infiltration. Additionally, SY reduced the number of *Bacilli* and *Alphaproteobacteria* and raised that of *Bacteroides* and *Erysipelotrichia*. It has been documented that *Bacteroides* is linked to several immunological and inflammatory systemic indicators [93]. These findings showed that SY may control the amount of gut microbiota linked to inflammation, which would control the tumor immune milieu. It is evident that SY has the ability to impact tumor immunology and intestinal microbiota, making it a viable option to increase the rate at which immunotherapy works.

#### Ganoderma lucidum

An important mushroom called *Ganoderma lucidum* (*G. lucidum*) has the potential to lower obesity in mice by altering the makeup of the microbiota in the gut and dramatically increase the activation of NK cells in patients with metastatic cancer [94, 95]. An extract obtained from *G. lucidum* (ESG) sporoderm-breaking spores shown immunoregulation and anticancer potential [96, 97]. The two immune checkpoints, CTLA-4 and PD-1, were both clearly downregulated by ESG. In the peripheral bloodstream in the tumor-bearing mouse, ESG also significantly increased the population of cytotoxic T cells (Tc) and the ratio of Tc to helper T cells (Th). These findings suggest that ESG could effectively restore the T cell paradigm from recuperating exhausted position through the suppression of co-inhibitory checkpoints. Additionally, ESG positively impacted the gut microbiome community, boosting the genera related to immunocompetence (*Helicobacter*, *Rikenella*, and *Turicibacter*) and decreasing some related to immunosuppression (Bacteroides), which may enhance CD8^+^ T cell-mediated immunity [34]. The root cause of ESG likely played a role in suppressing co-inhibitory signaling (PD-1 and CTLA-4) and restoring fatigued Tc cells, of which the gut microbiome remodeling was an important factor [98]. As a result, ESG can be utilized as a potential natural product for subsequent studies to adjust the gut microbiome and enhance the effectiveness of immune therapy by regulating the microbes. One of the most widely recognized *G. lucidum*-related compounds is oil of spores (GLSO). It was shown that GLSO can control the immune system by increasing NK cell cytotoxicity and macrophage phagocytosis in mice. A number of microbe genera and species, including *Lactobacillus*, *Turicibacter*, and *Romboutsia*, as well as *Lactobacillus intestinalis* and *Lactobacillus reuteri*, were found to be more abundant when GLSO was used. On the other hand, *Staphylococcus* and *Helicobacter* were found to be less abundant. These findings led to the regulation of a number of important metabolites, including dopamine, prolyl-glutamine, pentahomomethionine, leucyl-glutamine, L-threonine, stearoylcarnitine, and dolichyl β-D-glucosyl phosphate [99]. Therefore, GLSO can strengthen the immunological system of the body through the microbial metabolic axis, and is one of the potential natural products of anti-tumor immunotherapy.

#### Ginsenoside Rk3

With a lower molecular weight, *Ginsenoside Rk3* is produced from g*insenoside Rg1*. Previous research has demonstrated the antiapoptotic and anticancer effects of *ginsenoside Rk3* [100, 101]. *Ginsenoside Rk3* has been shown to be useful in establishing a healthy gut microbiota, which in turn can abrogate the invasion of gut macrophages, regulate immunological function, promote apoptosis in hepatocellular carcinoma, and prevent the growth of cancer. It effectively improved the amount of *Lactobacillus*, *Oscillibacter*, *Bacteroidetes*, *Lachnospiraceae*, *Bifidobacteriaceae*, *Akkermansia*, and *Bifidobacteriaceae* [102]. Additionally, it dramatically decreased the percentage of dangerous bacteria, including *Helicobacter*, *Ruminococcaceae*, and *Firmicutes*. Additionally, *ginsenoside Rk3* can downregulate inflammatory markers IL-17, IL-18, IL-22, and IL-6 linked to ILC3 and Th-17 [103], as well as directly boost the activities of *Akkermansia muciniphila* and glutamine concentration in *Akkermansia muciniphila* medium. Through immune system stimulation and gut microbiota regulation, it has anti-tumor effects. Additionally, as a possible natural product that modifies the microbes in the gut to improve the response of anti-tumor immunotherapy (Table 1).

**Table 1:** Natural products regulate DRTI through gut microbiota

Natural products	Agents	Cancer type	Targets	Effects of agents on gut microbiome	Immune regulation mechanism
*Soluble fiber*	*Pectin*	CRC	PD-1	*Lacto bacillaceae*, *Bifidobacteriaceae*, *Erysipelotrichaceae*, and *Ruminococcaceae*↑	Promote T cell infiltration and elicite CD8^+^ T cell
*Asteraceae*	*Inulin*	CRC	PD-1	*Akkermansia*, *Lactobacillus*, *Roseburia* hippurate, 1-methyl-L-histine, 3-methyl-L-histine, L-arginine, and oleate↑	Enhance the infiltration of CD8^+^ T cells and promote the differentiation of stem cells into Tcf1^+^PD-1^+^CD8^+^ T cells
*Jujube*	*Ultrafine jujube* powder (JP)	Colon cancer	PD-L1	*Lachnospiraceae*, *Lactobacillaceae*, *Ruminococcaceae*, SCFA↑	Increase CD8^+^ T-cell infiltration
*Bilberry*	*Anthocyanins*	Colon adenocarcinomas	PD-L1	*Clostridia* and *Lactobacillus johnsonii* and SCFAs↑	Enhanced CD8^+^ T cell infiltrations, butyrate↑
*Ginseng*	*Ginseng* polysaccharides	NSCLC	PD-1/ PD-L1	*B. vulgatus* and *P. distasonis*↑	Activating CD8^+^ T cells and suppressing the function of tregs
*Ginseng*	*Ginsenoside* Rk3	hepatocellular carcinoma	–	*Bacteroidetes*, *Lachnospiraceae*,* Bif idobacteriaceae*, *Akkermansia*, *Lactobacillus*, *Oscillibacter*, *Akkermansia muciniphila*↑; *Firmicutes*,* Helicobacter*,* Ruminococcaceae*↓	IL-17, IL-18, IL-22, and IL-6↓ glutamine↑
*Dioscorea*	*Diosgenin*	Melanoma	PD-1	*Lactobacillus genus, Sutterella genus*↑, *Bacteroides*↓	CD4^+^/CD8^+^ T-cell infiltration and IFN-γ expression↑
*Camu-camu*	*Castalagin*	Fibrosarcoma, mammary adenocarcinoma	PD-1	*Ruminococcus, Alistipes, Christensenellaceae R-7 *group and *Paraprevotella*↑ *Lachnosclostridium*↓	CD8^+^/CD4^+^FOXP3 T-cell ratio↑
*Gegen, Huangqin, Huanglian*	*Gegen Qinlian* decoction (GQD)	CRC	PD-1	*Bacteroidales Acidifaciens*↑	CD8^+^ T cells, IFN-γ↑
*Inonotus hispidus (Bull.: Fr.) P. Karst*	*Inonotus hispidus (Bull.: Fr.) P. Karst.* spore powder	CRC	–	*Oscillospira, Odoribacter, Rikenella, Dehalobacterium, Coprococcus* ↑ *Allobaculum, Mucispirillum*↓	Enhance CD8^+^ T cells infiltration
*Epimedium*	*Icariside* I	Melanoma	–	*Lactobacillus spp. *and *Bifidobacterium spp.*↑	CD4^+^ T, CD8^+^ T, NK, and NKT cells↑
*Carthamus tinctorius*	*Safflower yellow*	Liver tumour	–	*Bacteroides, Erysipelotrichia* ↑ *Bacilli, Alphaproteobacteria*↓	Enhance CD8^+^ T cells infiltration
*Ganoderma lucidum*	Sporoderm-breaking spores of *G. lucidum*	Breast cancer	PD-1 CTLA-4	*Helicobacter, Rikenella*, and *Turicibacte*↑, *Bacteroides*↓	Increase the Tc ratio and Tc/Th ratio
*Ganoderma lucidum*	The oil of *G. lucidum* spores	–	–	*Lactobacillus, Turicibacter, Romboutsia*↑ *Staphylococcus, Helicobacter*↓	macrophage phagocytosis, NK cell cytotoxicity↑
*Rheum officinale Baill., Prunus persica (L.) Batsch* and *Eupolyphaga sinensis Walker*	*Xiayuxue* decoction	Hepatocellular carcinoma	–	*Bacteroides and Lactobacillus*↑	CXCR6^+^NKT cell ↑
*Coix Seed, aconite, herba patriniae*	*Yi-Yi-Fu-Zi-Bai-Jiang-San*	CRC	–	*Bifidobacterium, Prevotellaceae, Lactobacillus, Ruminococcaceae, Clostridium, Lactobacillus rhamnosus* and* Clostridium butyricum*↑ *Bacteroides, Lachnospiraceae, unclassified lachnospiraceae, enterotoxigenic Bacteroides fragilis* mRNA↓	CD4^+^,CD25^+^ Foxp3 positive Treg cells, IL-6, IL-17A, RORγt, TNF-α↓
*Astragali Preparata, Atractylodes, Coicis Semen, Agrimonia pilosa Sparganii Rhizoma, Rhizoma Curcumae, Sophorae Flavescentis Radix, and Coptidis Rhizoma*	*Wenzi Jiedu* Recipe	CRC	–	*Oscillibacter, Bacteroides_acidifacien*↑ *Bacteroidales_bacterium*↓	CD8^+^ T cells, IL-10, IFN-g, and TNF-a↑
*Fuzi, Zhigancao,* and *Ganjiang*	*Sini* Decoction	CRC	–	*Bacillus coagulans, Lactobacillus, Bifidobacterium*↑ *Bacteroides fragilis, Sulphate reducing bacteria*↓	CD8^+^ T cells↑ CD4^+^ T cells, IL-6, IL-17, TNF-α, IFN-γ↓
*Medlar, raspberry, Epimedium, Psoralea, Duchesneau indica, dodder, Pinellia ternate, Radix puerariae, Radix scutellaria, mulberry, Prunella spica, Rattan pear root*, and *Rehmannia glutinosa*	Chinese herbal medicine anticancer cocktail soup	CRC	–	*Turicibacter, Faecalibaculum, Lactobacillus*↑, *Coriobacteriaceae_UCG002, Dubosiella, Lachnospiraceae_NK4A136, Lachnospiraceae*↓	Th17, CD8^+^ T cells, and NK cell↑

### Chinese native medicine compound prescription

#### Gegen Qinlian decoction

One widely recognized kind of classical TCM is *Gegen Qinlian decoction* (GQD). It can significantly lower inflammation and oxidative stress while also preventing kidney cancer in humans from developing [104–106]. Lv al. discovered that anti-mouse PD-1 combo therapy and GQD treatment significantly slowed the expansion of the tumor [107]. Additionally, the combination therapy markedly raised the percentage of CD8^+^ T cells and IFN-γ expression, both of which are essential components of anti-tumor immunotherapy. Combination therapy has also been shown to downregulate PD-1 and raise IL-2 levels, indicating that GQD may successfully reduce inhibitory checkpoints and rebuild T-cell activities. In addition to controlling the gut microbiome, GQD has been shown to enhance the number of LysoPC, vignatic acid B, LysoPE, LysoPE, and PI metabolites, all of which may strengthen the immune system. *Bacteroidales Acidifaciens* is one of the primary microbes that promotes IgA production in the intestinal tract [108, 109]. It was demonstrated that GQD controls the glycerophospholipid and sphingolipid metabolic pathways, amplifying the impact of PD-1 inhibition in CRC.

#### *Xiayuxue* decoction

*Prunus persica (L.) Batsch*, *Eupolyphaga sinensis Walker*, and *Rheum officinale Baill* are the three herbal products that make up the TCM compound formula known as *Xiayuxue* decoction (XYXD). Many pharmacological effects are demonstrated by XYXD, such as anti-tumor, anti-fibrosis, anti-inflammatory, gut microbiota regulation, and immune response modulation [110–112]. It was shown [113] that through modifying the relationship between gut microbiota and bile acids, XYXD can enhance the immunological impact of NKT cells against hepatocellular carcinoma (HCC). The primary process was augmenting the quantity of *Bacteroides* and *Lactobacillus* to facilitate the synthesis of bile salt hydrolase (BSH). By reducing the amount of *Eubacterium*, which lowers the transformation of main bile acids to secondary bile acids, BSH increases the level of primary bile acids and turns conjugated bile acids into primary bile acids. Primary bile acids cause the liver's NKT cells to release interferon-γ, which has anti-HCC immunological effects. Thus, by controlling the gut microbiota, XYXD is anticipated to be a potent natural medication ingredient that can improve the effectiveness of the immune system's response to tumors.

#### Yi-Yi-Fu-Zi-Bai-Jiang-San

A thousand-year-old *Huangdi Neijing* prescription known as *Yi-Yi-Fu-Zi-Bai-Jiang-San* (YYFZBJS) is frequently employed by TCM for treating problems with the digestive tract [114, 115]. It was discovered that YYFZBJS therapy for intestinal lymphatic and mesenteric lymph nodes (MLN) reduced the amount of accumulated CD4^+^ CD25^+^ Foxp3 positive treg cells in ApcMin/ + mice. The modified tregs caused by YYFZBJS also decreased the phosphorylation of β-catenin and inhibited the growth of CRC cancer cells. Subsequent research revealed that the complex microbiome is primarily responsible for this antitumor effect. Following YYFZBJS treatment, the prevalence of various probiotic genera significantly increased, including *Bifidobacterium* and *Prevotellaceae*, and nearly eliminated others, as *Bacteroides*, *Lachnospiraceae*, and Unclassified *Lachnospiraceae* [116]. Additionally, YYFZBJS can upregulate *Lactobacillus rhamnosus* (LGG) and *Clostridium* butyricum and raise the associated prevalence of pathogenic microbes, including *Lactobacillus*, *Ruminococcaceae*, and *Clostridium* [117]. Following YYFZBJS intervention, enterotoxigenic *Bacteroides fragilis* (ETBF) was shown to have reduced mRNA expression. Furthermore, YYFZBJS suppresses HIF-1α expression, enhances immune system response in *vivo* [118], causes treg cells to accumulate, modifies the function of nTreg cells that are naturally present, and significantly lowers the levels of the proteins IL-6, IL-17A, RORγt, and TNF-α. Consequently, YYFZBJS is a viable intervention tool for sensitizing and anti-tumor immunotherapy since it may simultaneously modulate gut microbiota and improve immune response.

#### Wenzi Jiedu recipe

Clinical evidence has demonstrated the efficacy of TCM formula Wenzi Jiedu recipe (WJR) in the treatment of CRC. Astragali Preparata, Atractylodes, Rhizoma Curcumae, Agrimonia pilosa Sparganii Rhizoma, Sophorae Flavescentis Radix, and Coptidis Rhizoma are among its contents. According to a study, WJR could regulate the immune system and gut microbiota to prevent the development of CRC [119]. WJR may, on the one hand, boost the percentage of CD8^+^ T cells and the production of TNF-a, IFN-g, and IL-10, three immune-associated cytokines. On the other hand, WJR may reduce harmful bacteria and raise helpful ones. Following WJR therapy, there is a drop in the amount of uncultured Bacteroides acidifacien and an increase in Oscillibacter and Bacteroides acidifacien. The anti-tumor immunotherapy response to WJR sensitization, however, has not been studied. WJR has the ability to stimulate the immune response to treat tumors through a variety of processes, including the modulation of the gut microbiome, the development of immune cells and immunologically linked proteins.

#### ***Sini*** decoction

*Fuzi*, *Zhigancao*, and *Ganjiang* are the three ingredients of *sini* decoction (SND), a traditional TCM remedy found in "*Shang Han Lun*." To treat CRC, SND extract can increase the expression of CD8^+^ T lymphocytes and occludin in the colonic mucosal layer while inhibiting the expression of CD4^+^ T cells and TNF-α, IL-6, IL-17, and IFN-γ in CRC tissue. Furthermore, SND can successfully interfere to the progression of CRC by modifying the composition of the gut microbiome by increasing the abundance of *Bifidobacterium*, *Lactobacillus*, and *Bacillus coagulans*, while decreasing *Bacteroides fragilis* other *Sulphateducing* microbes [120].

The composition of the intestinal microbiota in mice, enhanced by SND, influences the development and course of malignancies through immune system control. It may help to sensitize the immune system against cancers by regulating the gut microbiome.

#### Chinese herbal medicine anticancer cocktail soup

Prof. Te Liu of the *Shanghai* Geriatric Institute of Chinese Medicine and *Shanghai* University of Traditional Chinese Medicine created the Chinese herbal medicine anticancer cocktail soup (CHMACS), which was mostly made of extracts from 14 Chinese herbal products. CHMACS was shown to alter the gut microbiome-Th17 axis, which in turn activates immune cells and destroys cells. In CRC mice, CHMACS markedly increased Th17, CD8^+^, and NK cell activation, indicating increased immunological activity. In the intestinal microbiota of mice harboring tumors, it also boosted the abundance of *Turicibacter*, *Faecalibaculum*, and *Lactobacillus* while drastically lowering the amount of *Coriobacteriaceae*_UCG002, *Dubosiella*, *Lachnospiraceae*_NK4A136_group, and *Lachnospiraceae* [121]. Further research is necessary to determine the clinical use of CHMACS, which can improve anti-tumor immunity and control gut microbiota. This may play a therapeutic part in anti-tumor immunotherapy sensitization.

## Discussion

Across the past decade, there has been a notable advancement in the field of cancer immunotherapy. At the moment, PD-1/PD-L1 inhibitors and CTLA-4 inhibitors are receiving the greatest interest. These inhibitors mainly target B7/CTLA-4 and PD-1/PD-L1, two immunological checkpoints that are critical for T cell activation. Immune checkpoint inhibitors stimulate the differentiation and proliferation of T cells, improve the function of CD8^+^ T cells, activate the body's autoimmune function, and enhance the recognition and cytotoxicity function of dendritic cells on tumor cells or tissues by triggering natural killer (NK) cells in mononuclear macrophages. Maintain the tumor-immune cycle, activate immunological brake responses and successfully block tumor immune escap [122–127]. There are noteworthy differences in response rates among various cancer types, despite the fact that many individuals show a notable response and long-lasting effectiveness to immunotherapy. For instance, people with Hodgkin's lymphoma that is refractory may respond at a rate higher than 80%. On the other hand, patients with CRC that is proficient in mismatch repair show very little response [128, 129], and the total response rate in tumor immunotherapy varies from 20 to 40% [10]. Tumor immunotherapy's low response rate is therefore still a serious problem that needs to be solved.

The organism's intestinal microbiota is intimately connected to the immune system and is regarded as its second genome. Resistance to tumor immunotherapy may result from an imbalance in the gut microbiota and metabolites. Therefore, predicting the response of immune drugs effectively to avoid ineffective treatments and enhancing the response rate of patients to immunotherapy are major challenges in the field of tumor immunotherapy. It is well known that deviations in the WNT-β-catenin [130], MAPK [131], JAK/STAT [132], PI3K-AKT-mTOR [133], and Transforming growth factor-β signaling pathways are the primary causes of resistance to anti-tumor immunotherapy strategy [134]. And abnormalities also involve gene mutations in PTEN [135], SIRPA [136], TBK1 [137], the Y chromosome [138], as well as immune cells and stromal cells such as Treg [139], MDSCs [140], TAMs [141], CAFs [142], loss of tumor immunogenicity [143], abnormalities in Indoleamine 2,3-dioxygenase [144], Ubiquitin-specific protease 12 (USP12) [145], and Hypoxia [146]. Furthermore, it was found that the gut microbiota, which includes the bacteria *F. nucleatum*, *Coprobacillus cateniformis*, *Akkermansia muciniphila*, *Bifidobacterium*, *Ruminococcaceae*, *Faecalibacterium*, *Phyla Firmicutes*, and *Actinobacteria*, in addition to their metabolites, such as peptidoglycan hydrolase secretes antigen A, trimethylamine N-oxide, short-chain fatty acids, inosine, succinic acid, and L-arginine, are closely linked to the process of sensitizing the effectiveness of anti-tumor immunotherapy. In terms of treatment, *Pectin*, *Inulin*, *Jujube*, *Anthocyanins*, *Ginseng polysaccharides*, *Diosgenin*, *Camu-camu*, and *Inonotus hispidus* (*Bull.Fr. P. Karst*, *Icariside I*, *Safflower yellow*, *Ganoderma lucidum*, and *Ginsenoside Rk3* are natural active ingredients. Furthermore, a number of Chinese native medicine compound prescriptions have demonstrated impressive therapeutic results, including the *Sini* decoction, *Yi-Yi-Fu-Zi-Bai-Jiang-San*, *Gegen Qinlian* decoction, *Xiayuxue* decoction, *Wenzi Jiedu* recipe, and Chinese herbal medicine anticancer cocktail soup. Through the regulation of the microbiota and its metabolites, they may improve the effectiveness of anti-tumor immunotherapy.

The present study shows that natural products may alter the gut microbiome, which has important implications. In particular, these products decrease the frequency of dangerous bacteria, *Firmicutes*, *Helicobacter*, *Ruminococcaceae*, and others, while concurrently increasing the quantity of helpful probiotics, such as *Lactobacillaceae*, *Bifidobacteriaceae*, *Erysipelotrichaceae*, and *Ruminococcaceae*. Additionally, the study highlights the regulation of key intestinal microbial metabolites, such as butyrate, SCFAs, IDO, L-tryptophan, and L-kynurenine. These metabolites increase immune cell infiltration, which improves the system's reaction towards immunotherapy. Additionally, the research delineates plausible pathways via which natural compounds could potentially enhance the immune system's reaction to treatment. However, current research is mainly focused on CRC, NSCLC, hepatocellular carcinoma, melanoma, fibrosarcoma, and mammary adenocarcinoma, which has certain limitations. In addition, natural products exhibit multi-target and multi-pathway intervention effects. They enhance the efficacy of immunotherapy by regulating intestinal microbes and their metabolites. It did not, however, concentrate on the only regulatory mechanism, and there are no particular metabolites or microbes that can be used to forecast how well immunotherapy will work. Natural products are also expected to be considered as promising immunotherapy sensitizers for various malignant tumors, such as pancreatic cancer, esophageal cancer, and stomach cancer in the future.

Furthermore, we found that the natural active ingredients with the effect of invigorating *Qi* (*Jujube*, *Anthocyanins*, *Ginseng polysaccharides*, *Diosgenin*, *Icariside I*, *Ganoderma lucidum*, *Ginsenoside Rk3*) and promoting blood circulation(*Inonotus hispidus(Bull.: Fr.) P. Karst*, *safflower yellow* and *Xiayuxue* decoction) may be more dominant in the effect of sensitizing anti-tumor immunotherapy. The 2024 Nature Cancer study found [147] that co-stimulation of immune presenting cells and antigens associated with the tumor was essential for the substantial introduction of tumor-specific CD8^+^ T cells into the tumor microenvironment. This was determined by continuously monitoring the dynamic changes in the differentiation and proliferation of tumor-specific CD8^+^ T cells. There can be difficult to recruit CD8^+^ T cells into the tumor microenvironment if the antigen-presenting cells do not sufficiently convey the tumor antigen to the CD8^+^ T cells. As such, they are unable to multiply quickly enough to produce a potent anti-tumor reaction. Thus, the primary mechanism of immunotherapy resistance is a deficiency of T cells [148]. In TCM, *Qi* serves a physiological purpose akin to that of T cells in contemporary medicine. Their respective roles include controlling the body's immunity and defending against foreign infections. *Qi*-tonifying medications can boost T cell counts, which improves sensitization and encourages the immune system to fight cancer. A study that was published in Clinical Cancer Research in 2023 discovered [149] that heparin medication given in conjunction with anti-PD1 or adoptive cell transfer (ACT) produced combinatorial effects against cancer. These benefits have been attributed, possibly in a part, with tumor vasculature normalizing, which decreased the infiltration of treg while accelerating M1 macrophage polarization, ultimately leading to higher anticancer T-cell responses. Furthermore, a clinical trial [150] examining the combined use with the PD-1 immune checkpoint inhibitor carrilizumab and apatinib among individuals with advanced NSCLC who already received treatment with chemotherapy demonstrated a noteworthy clinical improvement for patients harboring STK11/KEAP1 mutations (The wild type, ORR is 28.1% and one-year survival is 53.1%; The mutation, ORR is 42.9% and one-year survival is 85.1%). The reason for the continuation of the Phase III trial (NCT04203485) may be because proper reduction of angiogenesis can improve PD-1/PD-L1 blocking through decreasing tumor-associated macrophage recruitment, boosting CD8^+^ T cell infiltration, and alleviating hypoxia. Blood-activating medications in traditional Chinese medicine are as effective as heparin and anti-angiogenic medications in contemporary medicine. Studies have indicated [151, 152] that natural products with blood-activating effects can facilitate the normalization of tumor blood vessels and inhibit tumor angiogenesis. Therefore, natural blood-activating drugs may enhance the response to anti-tumor immunotherapy by regulating tumor blood vessels.

The 2023 Cancer Cell study [153] demonstrated particular expression of flaky collagen and validated a substantial rise in fibroblasts after PD-1 therapy. In 2021, Nature study showed [154] that the discoidin domain receptor 1 (DDR1) performs a vital function in preventing immune cells in accessing the tumor site. By doing this, you can help surround the tumor with a physical barrier that prevents T cells for entering it and eliminating cancerous cells. Therefore, in addition to CD8^+^ cell depletion inhibiting the tumor immune response, immunotherapy induces a significant increase in tumor-associated fibroblasts, forming a spatial immune barrier to impede T cell infiltration. Although T cells are abundant, they are unable to penetrate the tumor microenvironment to carry out their functions, resulting in a diminished tumor immune response. Supplementing *Qi* and promoting blood circulation, as representative treatment methods in traditional Chinese medicine, may offer therapeutic benefits to patients with low responsiveness to T cell depletion immunotherapy by supporting qi supplementation and boosting immune function. By reducing metabolites and removing harmful gases, activating blood drugs may offer therapeutic benefits to patients with a low response to immunotherapy that inhibits T cell infiltration. However, the differences in natural products from various sources, types, and functions in primary and secondary drug resistance in anti-tumor immunotherapy still need further exploration.

In the future, studies on immunotherapy resistance should focus on predicting patients with primary and secondary resistance based on various factors, including demographic information, tumor stages, pathological types, biological indicators, and other relevant aspects. This approach will help identify the groups that will benefit most from immunotherapy, ultimately leading to more individualized and precise treatment [155]. Nevertheless, as the immune response's regulatory systems are intricate, the effectiveness and potential benefits to immunotherapy cannot be entirely predicted by simply one biomarker. Therefore, multiple biomarkers should be integrated in clinical applications. It is essential to develop a quantifiable predictive model for assessing the immune response. Utilizing a big data platform and leveraging artificial intelligence, aiming to construct a visual decision tree model characterized by high specificity, sensitivity, and accuracy, which will provide a reliable foundation for evaluating the predictive value of potential biomarkers [156]. In addition, biological samples such as pathological biopsies, peripheral blood, urine, and stool from immunoresistant patients can be obtained through the open biobank. By identifying particular targets and clarifying the mechanisms underlying immunoresistance [157, 158], new technologies such as single-cell sequencing and spatial ecology can open the door to the eventual clinical creation of new drugs.

Synthetic biology, medicine, and other fields are coming together to create novel drug delivery platforms and enhance existing medication processes, such as engineered bacteria, intestinal chips, and biofilm nanomedicine delivery systems, has gradually permeated clinical research and foundational studies on gut microbiota [46]. This advancement is driving progress in gut microbiota research, with the ultimate goal of identifying specific intestinal microorganisms or metabolites. It may boost the effectiveness of immunotherapy and turn patients with tumors who are refractory or secondary to tumor immunosuppressive therapy into patients who respond well to treatment. With the use of synthetic materials and modified microbes, immunotherapy may take a new turn in the future. Additionally [159], the application of small molecules from natural products and new polymer materials will also contribute to advancements in immunotherapy. The future trajectory for treating tumor immunodrug resistance may involve tailored multi-omics analysis in conjunction using personalized medicine, given the multiplicity of contributing factors and individual differences among the symbiotic microbiome.

## Data Availability

No data was used for the research described in the article.

## References

[1] Rowshanravan B, Halliday N, Sansom DM. CTLA-4: a moving target in immunotherapy. Blood. 2018;131(1):58–67. 29118008 10.1182/blood-2017-06-741033 PMC6317697

[2] Sun C, Mezzadra R, Schumacher TN. Regulation and function of the PD-L1 checkpoint. Immunity. 2018;48(3):434–52. 29562194 10.1016/j.immuni.2018.03.014 PMC7116507

[3] He X, Xu C. Immune checkpoint signaling and cancer immunotherapy. Cell Res. 2020;30(8):660–9. 32467592 10.1038/s41422-020-0343-4 PMC7395714

[4] Nathan P, Hassel JC, Rutkowski P, Baurain JF, Butler MO, Schlaak M, et al. Overall survival benefit with tebentafusp in metastatic uveal melanoma. N Engl J Med. 2021;385(13):1196–206. 34551229 10.1056/NEJMoa2103485

[5] Goldberg SB, Schalper KA, Gettinger SN, Mahajan A, Herbst RS, Chiang AC, et al. Pembrolizumab for management of patients with NSCLC and brain metastases: long-term results and biomarker analysis from a non-randomised, open-label, phase 2 trial. Lancet Oncol. 2020;21(5):655–63. 32251621 10.1016/S1470-2045(20)30111-X PMC7380514

[6] Xin YuJ, Hubbard-Lucey VM, Tang J. Immuno-oncology drug development goes global. Nat Rev Drug Discov. 2019;18(12):899–900. 31780841 10.1038/d41573-019-00167-9

[7] Robert C. A decade of immune-checkpoint inhibitors in cancer therapy. Nat Commun. 2020;11(1):3801. 32732879 10.1038/s41467-020-17670-y PMC7393098

[8] Ready NE, Ott PA, Hellmann MD, Zugazagoitia J, Hann CL, de Braud F, et al. Nivolumab monotherapy and nivolumab plus ipilimumab in recurrent small cell lung cancer: results from the CheckMate 032 randomized cohort. J Thorac Oncol. 2020;15(3):426–35. 31629915 10.1016/j.jtho.2019.10.004

[9] Bagchi S, Yuan R, Engleman EG. Immune checkpoint inhibitors for the treatment of cancer: clinical impact and mechanisms of response and resistance. Annu Rev Pathol. 2021;16:223–49. 33197221 10.1146/annurev-pathol-042020-042741

[10] Ribas A, Wolchok JD. Cancer immunotherapy using checkpoint blockade. Science. 2018;359(6382):1350–5. 29567705 10.1126/science.aar4060 PMC7391259

[11] Zhao B, Zhao H, Zhao J. Efficacy of PD-1/PD-L1 blockade monotherapy in clinical trials. Ther Adv Med Oncol. 2020;12:1758835920937612. 32728392 10.1177/1758835920937612 PMC7366397

[12] Garon EB, Rizvi NA, Hui R, Leighl N, Balmanoukian AS, Eder JP, et al. Pembrolizumab for the treatment of non-small-cell lung cancer. N Engl J Med. 2015;372(21):2018–28. 25891174 10.1056/NEJMoa1501824

[13] Ott PA, Bang YJ, Piha-Paul SA, Razak ARA, Bennouna J, Soria JC, et al. T-cell-inflamed gene-expression profile, programmed death ligand 1 expression, and tumor mutational burden predict efficacy in patients treated with pembrolizumab across 20 cancers: KEYNOTE-028. J Clin Oncol. 2019;37(4):318–27. 30557521 10.1200/JCO.2018.78.2276

[14] Jugder BE, Kamareddine L, Watnick PI. Microbiota-derived acetate activates intestinal innate immunity via the Tip60 histone acetyltransferase complex. Immunity. 2021;54(8):1683-97 e3. 34107298 10.1016/j.immuni.2021.05.017 PMC8363570

[15] Zheng D, Liwinski T, Elinav E. Interaction between microbiota and immunity in health and disease. Cell Res. 2020;30(6):492–506. 32433595 10.1038/s41422-020-0332-7 PMC7264227

[16] Miyauchi E, Kim SW, Suda W, Kawasumi M, Onawa S, Taguchi-Atarashi N, et al. Gut microorganisms act together to exacerbate inflammation in spinal cords. Nature. 2020;585(7823):102–6. 32848245 10.1038/s41586-020-2634-9

[17] Erny D, Dokalis N, Mezo C, Castoldi A, Mossad O, Staszewski O, et al. Microbiota-derived acetate enables the metabolic fitness of the brain innate immune system during health and disease. Cell Metab. 2021;33(11):2260-76 e7. 34731656 10.1016/j.cmet.2021.10.010

[18] Morais LH, Schreiber HLT, Mazmanian SK. The gut microbiota-brain axis in behaviour and brain disorders. Nat Rev Microbiol. 2021;19(4):241–55. 33093662 10.1038/s41579-020-00460-0

[19] He Y, Fu L, Li Y, Wang W, Gong M, Zhang J, et al. Gut microbial metabolites facilitate anticancer therapy efficacy by modulating cytotoxic CD8(+) T cell immunity. Cell Metab. 2021;33(5):988-1000 e7. 33761313 10.1016/j.cmet.2021.03.002

[20] Microbiota-induced IF. I signaling promotes an antitumor microenvironment. Cancer Discov. 2021;11(12):2955. 10.1158/2159-8290.CD-RW2021-145 34654702

[21] Stower H. Microbiome transplant-induced response to immunotherapy. Nat Med. 2021;27(1):21. 33442010 10.1038/s41591-020-01220-6

[22] McQuade JL, Daniel CR, Helmink BA, Wargo JA. Modulating the microbiome to improve therapeutic response in cancer. Lancet Oncol. 2019;20(2):e77–91. 30712808 10.1016/S1470-2045(18)30952-5 PMC12908161

[23] Fehervari Z. Microbiota shape tumor immunity. Nat Immunol. 2021;22(12):1469. 34811539 10.1038/s41590-021-01082-1

[24] Chen YC, He XL, Qi L, Shi W, Yuan LW, Huang MY, et al. Myricetin inhibits interferon-gamma-induced PD-L1 and IDO1 expression in lung cancer cells. Biochem Pharmacol. 2022;197: 114940. 35120895 10.1016/j.bcp.2022.114940

[25] Hu Q, Jiang L, Yan Q, Zeng J, Ma X, Zhao Y. A natural products solution to diabetic nephropathy therapy. Pharmacol Ther. 2023;241: 108314. 36427568 10.1016/j.pharmthera.2022.108314

[26] Duan S, Zhang M, Zeng H, Song J, Zhang M, Gao S, et al. Integrated proteomics and phosphoproteomics profiling reveals the cardioprotective mechanism of bioactive compounds derived from Salvia miltiorrhiza Burge. Phytomedicine. 2023;117: 154897. 37307738 10.1016/j.phymed.2023.154897

[27] Peng Z, Cheng S, Kou Y, Wang Z, Jin R, Hu H, et al. The gut microbiome is associated with clinical response to Anti-PD-1/PD-L1 immunotherapy in gastrointestinal cancer. Cancer Immunol Res. 2020;8(10):1251–61. 32855157 10.1158/2326-6066.CIR-19-1014

[28] Fenton TM, Jorgensen PB, Niss K, Rubin SJS, Morbe UM, Riis LB, et al. Immune profiling of human gut-associated lymphoid tissue identifies a role for isolated lymphoid follicles in priming of region-specific immunity. Immunity. 2020;52(3):557-70 e6. 32160523 10.1016/j.immuni.2020.02.001 PMC7155934

[29] Jiang SS, Xie YL, Xiao XY, Kang ZR, Lin XL, Zhang L, et al. Fusobacterium nucleatum-derived succinic acid induces tumor resistance to immunotherapy in colorectal cancer. Cell Host Microbe. 2023;31(5):781-97 e9. 37130518 10.1016/j.chom.2023.04.010

[30] Park JS, Gazzaniga FS, Wu M, Luthens AK, Gillis J, Zheng W, et al. Targeting PD-L2-RGMb overcomes microbiome-related immunotherapy resistance. Nature. 2023;617(7960):377–85. 37138075 10.1038/s41586-023-06026-3 PMC10219577

[31] Routy B, Le Chatelier E, Derosa L, Duong CPM, Alou MT, Daillere R, et al. Gut microbiome influences efficacy of PD-1-based immunotherapy against epithelial tumors. Science. 2018;359(6371):91–7. 29097494 10.1126/science.aan3706

[32] Derosa L, Routy B, Fidelle M, Iebba V, Alla L, Pasolli E, et al. Gut bacteria composition drives primary resistance to cancer immunotherapy in renal cell carcinoma patients. Eur Urol. 2020;78(2):195–206. 32376136 10.1016/j.eururo.2020.04.044

[33] Matson V, Fessler J, Bao R, Chongsuwat T, Zha Y, Alegre ML, et al. The commensal microbiome is associated with anti-PD-1 efficacy in metastatic melanoma patients. Science. 2018;359(6371):104–8. 29302014 10.1126/science.aao3290 PMC6707353

[34] Sivan A, Corrales L, Hubert N, Williams JB, Aquino-Michaels K, Earley ZM, et al. Commensal Bifidobacterium promotes antitumor immunity and facilitates anti-PD-L1 efficacy. Science. 2015;350(6264):1084–9. 26541606 10.1126/science.aac4255 PMC4873287

[35] Gopalakrishnan V, Spencer CN, Nezi L, Reuben A, Andrews MC, Karpinets TV, et al. Gut microbiome modulates response to anti-PD-1 immunotherapy in melanoma patients. Science. 2018;359(6371):97–103. 29097493 10.1126/science.aan4236 PMC5827966

[36] Chaput N, Lepage P, Coutzac C, Soularue E, Le Roux K, Monot C, et al. Baseline gut microbiota predicts clinical response and colitis in metastatic melanoma patients treated with ipilimumab. Ann Oncol. 2017;28(6):1368–79. 28368458 10.1093/annonc/mdx108

[37] Davar D, Dzutsev AK, McCulloch JA, Rodrigues RR, Chauvin JM, Morrison RM, et al. Fecal microbiota transplant overcomes resistance to anti-PD-1 therapy in melanoma patients. Science. 2021;371(6529):595–602. 33542131 10.1126/science.abf3363 PMC8097968

[38] Griffin ME, Espinosa J, Becker JL, Luo JD, Carroll TS, Jha JK, et al. Enterococcus peptidoglycan remodeling promotes checkpoint inhibitor cancer immunotherapy. Science. 2021;373(6558):1040–6. 34446607 10.1126/science.abc9113 PMC9503018

[39] Wang H, Rong X, Zhao G, Zhou Y, Xiao Y, Ma D, et al. The microbial metabolite trimethylamine N-oxide promotes antitumor immunity in triple-negative breast cancer. Cell Metab. 2022;34(4):581-94 e8. 35278352 10.1016/j.cmet.2022.02.010

[40] Mirji G, Worth A, Bhat SA, El Sayed M, Kannan T, Goldman AR, et al. The microbiome-derived metabolite TMAO drives immune activation and boosts responses to immune checkpoint blockade in pancreatic cancer. Sci Immunol. 2022;7(75):eabn0704. 36083892 10.1126/sciimmunol.abn0704 PMC9925043

[41] Coutzac C, Jouniaux JM, Paci A, Schmidt J, Mallardo D, Seck A, et al. Systemic short chain fatty acids limit antitumor effect of CTLA-4 blockade in hosts with cancer. Nat Commun. 2020;11(1):2168. 32358520 10.1038/s41467-020-16079-x PMC7195489

[42] Luu M, Visekruna A. Microbial metabolites: novel therapeutic tools for boosting cancer therapies. Trends Cell Biol. 2021;31(11):873–5. 34538658 10.1016/j.tcb.2021.08.005

[43] Zhang L, Jiang L, Yu L, Li Q, Tian X, He J, et al. Inhibition of UBA6 by inosine augments tumour immunogenicity and responses. Nat Commun. 2022;13(1):5413. 36109526 10.1038/s41467-022-33116-z PMC9478149

[44] Patil MD, Bhaumik J, Babykutty S, Banerjee UC, Fukumura D. Arginine dependence of tumor cells: targeting a chink in cancer’s armor. Oncogene. 2016;35(38):4957–72. 27109103 10.1038/onc.2016.37 PMC5457742

[45] He X, Lin H, Yuan L, Li B. Combination therapy with L-arginine and alpha-PD-L1 antibody boosts immune response against osteosarcoma in immunocompetent mice. Cancer Biol Ther. 2017;18(2):94–100. 28045576 10.1080/15384047.2016.1276136 PMC5362985

[46] Canale FP, Basso C, Antonini G, Perotti M, Li N, Sokolovska A, et al. Metabolic modulation of tumours with engineered bacteria for immunotherapy. Nature. 2021;598(7882):662–6. 34616044 10.1038/s41586-021-04003-2

[47] Friedlaender A, Addeo A, Banna G. New emerging targets in cancer immunotherapy: the role of TIM3. ESMO Open. 2019;4(Suppl 3): e000497. 31275616 10.1136/esmoopen-2019-000497 PMC6579568

[48] Qiu H, Shao Z, Wen X, Jiang J, Ma Q, Wang Y, et al. TREM2: keeping pace with immune checkpoint inhibitors in cancer immunotherapy. Front Immunol. 2021;12: 716710. 34539652 10.3389/fimmu.2021.716710 PMC8446424

[49] Feng X, Li Z, Guo W, Hu Y. The effects of traditional Chinese medicine and dietary compounds on digestive cancer immunotherapy and gut microbiota modulation: a review. Front Immunol. 2023;14:1087755. 36845103 10.3389/fimmu.2023.1087755 PMC9945322

[50] Annunziata G, Maisto M, Schisano C, Ciampaglia R, Narciso V, Hassan STS, et al. Effect of grape pomace polyphenols with or without pectin on TMAO serum levels assessed by LC/MS-based assay: a preliminary clinical study on overweight/obese subjects. Front Pharmacol. 2019;10:575. 31164827 10.3389/fphar.2019.00575 PMC6536651

[51] Zhang SL, Mao YQ, Zhang ZY, Li ZM, Kong CY, Chen HL, et al. Pectin supplement significantly enhanced the anti-PD-1 efficacy in tumor-bearing mice humanized with gut microbiota from patients with colorectal cancer. Theranostics. 2021;11(9):4155–70. 33754054 10.7150/thno.54476 PMC7977465

[52] Zhang T, Chi Z, Zhao CH, Chi ZM, Gong F. Bioethanol production from hydrolysates of inulin and the tuber meal of Jerusalem artichoke by Saccharomyces sp. W0. Bioresour Technol. 2010;101(21):8166–70. 20598527 10.1016/j.biortech.2010.06.013

[53] Samanta AK, Jayapal N, Senani S, Kolte AP, Sridhar M. Prebiotic inulin: useful dietary adjuncts to manipulate the livestock gut microflora. Braz J Microbiol. 2013;44(1):1–14. 24159277 10.1590/S1517-83822013005000023 PMC3804171

[54] Han K, Nam J, Xu J, Sun X, Huang X, Animasahun O, et al. Generation of systemic antitumour immunity via the in situ modulation of the gut microbiome by an orally administered inulin gel. Nat Biomed Eng. 2021;5(11):1377–88. 34168321 10.1038/s41551-021-00749-2 PMC8595497

[55] Stewart ML, Savarino V, Slavin JL. Assessment of dietary fiber fermentation: effect of Lactobacillus reuteri and reproducibility of short-chain fatty acid concentrations. Mol Nutr Food Res. 2009;53(Suppl 1):S114–20. 18837468 10.1002/mnfr.200700523

[56] La Rosa SL, Leth ML, Michalak L, Hansen ME, Pudlo NA, Glowacki R, et al. The human gut Firmicute Roseburia intestinalis is a primary degrader of dietary beta-mannans. Nat Commun. 2019;10(1):905. 30796211 10.1038/s41467-019-08812-y PMC6385246

[57] Hatae R, Chamoto K, Kim YH, Sonomura K, Taneishi K, Kawaguchi S, et al. Combination of host immune metabolic biomarkers for the PD-1 blockade cancer immunotherapy. JCI Insight. 2020. 10.1172/jci.insight.133501. 31855576 10.1172/jci.insight.133501 PMC7098729

[58] Choi SH, Ahn JB, Kozukue N, Levin CE, Friedman M. Distribution of free amino acids, flavonoids, total phenolics, and antioxidative activities of Jujube (Ziziphus jujuba) fruits and seeds harvested from plants grown in Korea. J Agric Food Chem. 2011;59(12):6594–604. 21574660 10.1021/jf200371r

[59] Daneshmand F, Zare-Zardini H, Ebrahimi L. Investigation of the antimicrobial activities of Snakin-Z, a new cationic peptide derived from Zizyphus jujuba fruits. Nat Prod Res. 2013;27(24):2292–6. 23962183 10.1080/14786419.2013.827192

[60] Zhou Y, Li Y, Zhou T, Zheng J, Li S, Li HB. Dietary natural products for prevention and treatment of liver cancer. Nutrients. 2016;8(3):156. 26978396 10.3390/nu8030156 PMC4808884

[61] Li J, Shan L, Liu Y, Fan L, Ai L. Screening of a functional polysaccharide from Zizyphus Jujuba cv. Jinsixiaozao and its property. Int J Biol Macromol. 2011;49(3):255–9. 21539856 10.1016/j.ijbiomac.2011.04.006

[62] Zou M, Chen Y, Sun-Waterhouse D, Zhang Y, Li F. Immunomodulatory acidic polysaccharides from Zizyphus jujuba cv. Huizao: insights into their chemical characteristics and modes of action. Food Chem. 2018;258:35–42. 29655744 10.1016/j.foodchem.2018.03.052

[63] Jing N, Wang L, Zhuang H, Jiang G, Liu Z. Ultrafine Jujube Powder Enhances the Infiltration of Immune Cells during Anti-PD-L1 Treatment against Murine Colon Adenocarcinoma. Cancers (Basel). 2021. 10.3390/cancers13163987. 34439144 10.3390/cancers13163987 PMC8394940

[64] Tanoue T, Morita S, Plichta DR, Skelly AN, Suda W, Sugiura Y, et al. A defined commensal consortium elicits CD8 T cells and anti-cancer immunity. Nature. 2019;565(7741):600–5. 30675064 10.1038/s41586-019-0878-z

[65] Mager LF, Burkhard R, Pett N, Cooke NCA, Brown K, Ramay H, et al. Microbiome-derived inosine modulates response to checkpoint inhibitor immunotherapy. Science. 2020;369(6510):1481–9. 32792462 10.1126/science.abc3421

[66] Lala G, Malik M, Zhao C, He J, Kwon Y, Giusti MM, et al. Anthocyanin-rich extracts inhibit multiple biomarkers of colon cancer in rats. Nutr Cancer. 2006;54(1):84–93. 16800776 10.1207/s15327914nc5401_10

[67] Hui C, Bin Y, Xiaoping Y, Long Y, Chunye C, Mantian M, et al. Anticancer activities of an anthocyanin-rich extract from black rice against breast cancer cells in vitro and in vivo. Nutr Cancer. 2010;62(8):1128–36. 21058201 10.1080/01635581.2010.494821

[68] Correa-Betanzo J, Allen-Vercoe E, McDonald J, Schroeter K, Corredig M, Paliyath G. Stability and biological activity of wild blueberry (Vaccinium angustifolium) polyphenols during simulated in vitro gastrointestinal digestion. Food Chem. 2014;165:522–31. 25038707 10.1016/j.foodchem.2014.05.135

[69] Wang L, Jiang G, Jing N, Liu X, Li Q, Liang W, et al. Bilberry anthocyanin extracts enhance anti-PD-L1 efficiency by modulating gut microbiota. Food Funct. 2020;11(4):3180–90. 32211663 10.1039/d0fo00255k

[70] Ohira H, Tsutsui W, Fujioka Y. Are short chain fatty acids in gut microbiota defensive players for inflammation and atherosclerosis? J Atheroscler Thromb. 2017;24(7):660–72. 28552897 10.5551/jat.RV17006 PMC5517538

[71] Hansen JJ. Immune responses to intestinal microbes in inflammatory bowel diseases. Curr Allergy Asthma Rep. 2015;15(10):61. 26306907 10.1007/s11882-015-0562-9

[72] Liu X, Wang L, Jing N, Jiang G, Liu Z. Biostimulating gut microbiome with bilberry anthocyanin combo to enhance anti-PD-L1 efficiency against murine colon cancer. Microorganisms. 2020. 10.3390/microorganisms8020175. 31991820 10.3390/microorganisms8020175 PMC7074734

[73] Yang SH, Seo SH, Kim SW, Choi SK, Kim DH. Effect of ginseng polysaccharide on the stability of lactic acid bacteria during freeze-drying process and storage. Arch Pharm Res. 2006;29(9):735–40. 17024845 10.1007/BF02974072

[74] Huang J, Liu D, Wang Y, Liu L, Li J, Yuan J, et al. Ginseng polysaccharides alter the gut microbiota and kynurenine/tryptophan ratio, potentiating the antitumour effect of antiprogrammed cell death 1/programmed cell death ligand 1 (anti-PD-1/PD-L1) immunotherapy. Gut. 2022;71(4):734–45. 34006584 10.1136/gutjnl-2020-321031 PMC8921579

[75] Jan TR, Wey SP, Kuan CC, Liao MH, Wu HY. Diosgenin, a steroidal sapogenin, enhances antigen-specific IgG2a and interferon-gamma expression in ovalbumin-sensitized BALB/c mice. Planta Med. 2007;73(5):421–6. 17566144 10.1055/s-2007-967169

[76] Raju J, Mehta R. Cancer chemopreventive and therapeutic effects of diosgenin, a food saponin. Nutr Cancer. 2009;61(1):27–35. 19116873 10.1080/01635580802357352

[77] Dong M, Meng Z, Kuerban K, Qi F, Liu J, Wei Y, et al. Diosgenin promotes antitumor immunity and PD-1 antibody efficacy against melanoma by regulating intestinal microbiota. Cell Death Dis. 2018;9(10):1039. 30305604 10.1038/s41419-018-1099-3 PMC6179990

[78] Anhe FF, Nachbar RT, Varin TV, Trottier J, Dudonne S, Le Barz M, et al. Treatment with camu camu (Myrciaria dubia) prevents obesity by altering the gut microbiota and increasing energy expenditure in diet-induced obese mice. Gut. 2019;68(3):453–64. 30064988 10.1136/gutjnl-2017-315565

[79] Messaoudene M, Pidgeon R, Richard C, Ponce M, Diop K, Benlaifaoui M, et al. A natural polyphenol exerts antitumor activity and circumvents Anti-PD-1 resistance through effects on the gut microbiota. Cancer Discov. 2022;12(4):1070–87. 35031549 10.1158/2159-8290.CD-21-0808 PMC9394387

[80] van Heumen BW, Roelofs HM, Te Morsche RH, Marian B, Nagengast FM, Peters WH. Celecoxib and tauro-ursodeoxycholic acid co-treatment inhibits cell growth in familial adenomatous polyposis derived LT97 colon adenoma cells. Exp Cell Res. 2012;318(7):819–27. 22366264 10.1016/j.yexcr.2012.02.004

[81] Sari A, Tuzen M. Biosorption of As(III) and As(V) from aqueous solution by macrofungus (Inonotus hispidus) biomass: equilibrium and kinetic studies. J Hazard Mater. 2009;164(2–3):1372–8. 19022572 10.1016/j.jhazmat.2008.09.047

[82] Wang ZX, Feng XL, Liu C, Gao JM, Qi J. Diverse metabolites and pharmacological effects from the basidiomycetes inonotus hispidus. Antibiotics (Basel). 2022. 10.3390/antibiotics11081097. 36009965 10.3390/antibiotics11081097 PMC9405263

[83] Yang H, Li S, Qu Y, Li L, Li Y, Wang D. Anti-colorectal cancer effects of inonotus hispidus (Bull.: Fr.) P. karst spore powder through regulation of gut microbiota-mediated JAK/STAT signaling. Nutrients. 2022. 10.3390/nu14163299. 36014805 10.3390/nu14163299 PMC9415721

[84] Bronte V, Serafini P, Mazzoni A, Segal DM, Zanovello P. L-arginine metabolism in myeloid cells controls T-lymphocyte functions. Trends Immunol. 2003;24(6):302–6. 12810105 10.1016/s1471-4906(03)00132-7

[85] Zhang DW, Cheng Y, Wang NL, Zhang JC, Yang MS, Yao XS. Effects of total flavonoids and flavonol glycosides from Epimedium koreanum Nakai on the proliferation and differentiation of primary osteoblasts. Phytomedicine. 2008;15(1–2):55–61. 17482445 10.1016/j.phymed.2007.04.002

[86] Huang X, Zhu D, Lou Y. A novel anticancer agent, icaritin, induced cell growth inhibition, G1 arrest and mitochondrial transmembrane potential drop in human prostate carcinoma PC-3 cells. Eur J Pharmacol. 2007;564(1–3):26–36. 17382317 10.1016/j.ejphar.2007.02.039

[87] Chen G, Cao Z, Shi Z, Lei H, Chen C, Yuan P, et al. Microbiome analysis combined with targeted metabolomics reveal immunological anti-tumor activity of icariside I in a melanoma mouse model. Biomed Pharmacother. 2021;140: 111542. 34088571 10.1016/j.biopha.2021.111542

[88] Hu J, Wang C, Ye L, Yang W, Huang H, Meng F, et al. Anti-tumour immune effect of oral administration of Lactobacillus plantarum to CT26 tumour-bearing mice. J Biosci. 2015;40(2):269–79. 25963256 10.1007/s12038-015-9518-4

[89] Ma X, Zhou Z, Zhang X, Fan M, Hong Y, Feng Y, et al. Sodium butyrate modulates gut microbiota and immune response in colorectal cancer liver metastatic mice. Cell Biol Toxicol. 2020;36(5):509–15. 32172331 10.1007/s10565-020-09518-4

[90] Singh N, Gurav A, Sivaprakasam S, Brady E, Padia R, Shi H, et al. Activation of Gpr109a, receptor for niacin and the commensal metabolite butyrate, suppresses colonic inflammation and carcinogenesis. Immunity. 2014;40(1):128–39. 24412617 10.1016/j.immuni.2013.12.007 PMC4305274

[91] Fu H, Wu R, Li Y, Zhang L, Tang X, Tu J, et al. Safflower yellow prevents pulmonary metastasis of breast cancer by inhibiting tumor cell invadopodia. Am J Chin Med. 2016;44(7):1491–506. 27776431 10.1142/S0192415X1650083X

[92] Fu H, Liu X, Jin L, Lang J, Hu Z, Mao W, et al. Safflower yellow reduces DEN-induced hepatocellular carcinoma by enhancing liver immune infiltration through promotion of collagen degradation and modulation of gut microbiota. Food Funct. 2021;12(21):10632–43. 34585698 10.1039/d1fo01321a

[93] Liu Q, Li F, Zhuang Y, Xu J, Wang J, Mao X, et al. Alteration in gut microbiota associated with hepatitis B and non-hepatitis virus related hepatocellular carcinoma. Gut Pathog. 2019;11:1. 30675188 10.1186/s13099-018-0281-6 PMC6337822

[94] Gao Y, Zhou S, Jiang W, Huang M, Dai X. Effects of ganopoly (a Ganoderma lucidum polysaccharide extract) on the immune functions in advanced-stage cancer patients. Immunol Invest. 2003;32(3):201–15. 12916709 10.1081/imm-120022979

[95] Chang CJ, Lin CS, Lu CC, Martel J, Ko YF, Ojcius DM, et al. Ganoderma lucidum reduces obesity in mice by modulating the composition of the gut microbiota. Nat Commun. 2015;6:7489. 26102296 10.1038/ncomms8489 PMC4557287

[96] Na K, Li K, Sang T, Wu K, Wang Y, Wang X. Anticarcinogenic effects of water extract of sporoderm-broken spores of Ganoderma lucidum on colorectal cancer in vitro and in vivo. Int J Oncol. 2017;50(5):1541–54. 28358412 10.3892/ijo.2017.3939 PMC5403400

[97] Yue GG, Fung KP, Leung PC, Lau CB. Comparative studies on the immunomodulatory and antitumor activities of the different parts of fruiting body of Ganoderma lucidum and Ganoderma spores. Phytother Res. 2008;22(10):1282–91. 18570198 10.1002/ptr.2478

[98] Su J, Su L, Li D, Shuai O, Zhang Y, Liang H, et al. Antitumor activity of extract from the sporoderm-breaking spore of Ganoderma lucidum: restoration on exhausted cytotoxic T Cell with gut microbiota remodeling. Front Immunol. 2018;9:1765. 30108589 10.3389/fimmu.2018.01765 PMC6079217

[99] Wu X, Cao J, Li M, Yao P, Li H, Xu W, et al. An integrated microbiome and metabolomic analysis identifies immunoenhancing features of Ganoderma lucidum spores oil in mice. Pharmacol Res. 2020;158: 104937. 32464331 10.1016/j.phrs.2020.104937

[100] Chen H, Yang H, Deng J, Fan D. Ginsenoside Rk3 ameliorates obesity-induced colitis by regulating of intestinal flora and the TLR4/NF-kappaB signaling pathway in C57BL/6 Mice. J Agric Food Chem. 2021;69(10):3082–93. 33621094 10.1021/acs.jafc.0c07805

[101] Liu Y, Fan D. Ginsenoside Rg5 induces apoptosis and autophagy via the inhibition of the PI3K/Akt pathway against breast cancer in a mouse model. Food Funct. 2018;9(11):5513–27. 30207362 10.1039/c8fo01122b

[102] Qu L, Ma X, Fan D. Ginsenoside Rk3 suppresses hepatocellular carcinoma development through targeting the gut-liver axis. J Agric Food Chem. 2021;69(35):10121–37. 34415764 10.1021/acs.jafc.1c03279

[103] Bai X, Fu R, Liu Y, Deng J, Fei Q, Duan Z, et al. Ginsenoside Rk3 modulates gut microbiota and regulates immune response of group 3 innate lymphoid cells to against colorectal tumorigenesis. J Pharm Anal. 2024;14(2):259–75. 38464791 10.1016/j.jpha.2023.09.010 PMC10921328

[104] Wang N, Feng Y, Cheung F, Wang X, Zhang Z, Feng Y. A Chinese medicine formula Gegen Qinlian decoction suppresses expansion of human renal carcinoma with inhibition of matrix metalloproteinase-2. Integr Cancer Ther. 2015;14(1):75–85. 25228536 10.1177/1534735414550036

[105] Cui L, Feng L, Zhang ZH, Jia XB. The anti-inflammation effect of baicalin on experimental colitis through inhibiting TLR4/NF-kappaB pathway activation. Int Immunopharmacol. 2014;23(1):294–303. 25239813 10.1016/j.intimp.2014.09.005

[106] Lee IA, Hyun YJ, Kim DH. Berberine ameliorates TNBS-induced colitis by inhibiting lipid peroxidation, enterobacterial growth and NF-kappaB activation. Eur J Pharmacol. 2010;648(1–3):162–70. 20828550 10.1016/j.ejphar.2010.08.046

[107] Lv J, Jia Y, Li J, Kuai W, Li Y, Guo F, et al. Gegen Qinlian decoction enhances the effect of PD-1 blockade in colorectal cancer with microsatellite stability by remodelling the gut microbiota and the tumour microenvironment. Cell Death Dis. 2019;10(6):415. 31138779 10.1038/s41419-019-1638-6 PMC6538740

[108] Yanagibashi T, Hosono A, Oyama A, Tsuda M, Suzuki A, Hachimura S, et al. IgA production in the large intestine is modulated by a different mechanism than in the small intestine: Bacteroides acidifaciens promotes IgA production in the large intestine by inducing germinal center formation and increasing the number of IgA+ B cells. Immunobiology. 2013;218(4):645–51. 22940255 10.1016/j.imbio.2012.07.033

[109] Palm NW, de Zoete MR, Cullen TW, Barry NA, Stefanowski J, Hao L, et al. Immunoglobulin A coating identifies colitogenic bacteria in inflammatory bowel disease. Cell. 2014;158(5):1000–10. 25171403 10.1016/j.cell.2014.08.006 PMC4174347

[110] Ji G, Ma L, Yao H, Ma S, Si X, Wang Y, et al. Precise delivery of obeticholic acid via nanoapproach for triggering natural killer T cell-mediated liver cancer immunotherapy. Acta Pharm Sin B. 2020;10(11):2171–82. 33304784 10.1016/j.apsb.2020.09.004 PMC7715527

[111] Ji C, Deng Y, Yang A, Lu Z, Chen Y, Liu X, et al. Rhubarb enema improved colon mucosal barrier injury in 5/6 nephrectomy rats may associate with gut microbiota modification. Front Pharmacol. 2020;11:1092. 32848732 10.3389/fphar.2020.01092 PMC7403201

[112] Jiang H, Tang W, Song Y, Jin W, Du Q. Induction of apoptosis by metabolites of Rhei Radix et Rhizoma (Da Huang): a review of the potential mechanism in hepatocellular carcinoma. Front Pharmacol. 2022;13: 806175. 35308206 10.3389/fphar.2022.806175 PMC8924367

[113] Deng Z, Ouyang Z, Mei S, Zhang X, Li Q, Meng F, et al. Enhancing NKT cell-mediated immunity against hepatocellular carcinoma: Role of XYXD in promoting primary bile acid synthesis and improving gut microbiota. J Ethnopharmacol. 2024;318(Pt B): 116945. 37490989 10.1016/j.jep.2023.116945

[114] Guo M, Ding S, Zhao C, Gu X, He X, Huang K, et al. Red Ginseng and Semen Coicis can improve the structure of gut microbiota and relieve the symptoms of ulcerative colitis. J Ethnopharmacol. 2015;162:7–13. 25554637 10.1016/j.jep.2014.12.029

[115] Xia L, Zhang B, Yan Q, Ruan S. Effects of saponins of patrinia villosa against invasion and metastasis in colorectal cancer cell through NF-kappaB signaling pathway and EMT. Biochem Biophys Res Commun. 2018;503(3):2152–9. 30119890 10.1016/j.bbrc.2018.08.005

[116] Sui H, Zhang L, Gu K, Chai N, Ji Q, Zhou L, et al. YYFZBJS ameliorates colorectal cancer progression in Apc(Min/+) mice by remodeling gut microbiota and inhibiting regulatory T-cell generation. Cell Commun Signal. 2020;18(1):113. 32677955 10.1186/s12964-020-00596-9 PMC7367414

[117] Chai N, Xiong Y, Zhang Y, Cheng Y, Shi W, Yao Y, et al. YYFZBJS inhibits colorectal tumorigenesis by remodeling gut microbiota and influence on M2 macrophage polarization in vivo and in vitro. Am J Cancer Res. 2021;11(11):5338–57. 34873464 PMC8640793

[118] Zhang Y, Chai N, Wei Z, Li Z, Zhang L, Zhang M, et al. YYFZBJS inhibits colorectal tumorigenesis by enhancing Tregs-induced immunosuppression through HIF-1alpha mediated hypoxia in vivo and in vitro. Phytomedicine. 2022;98: 153917. 35093671 10.1016/j.phymed.2021.153917

[119] Qiu W, Sang T, Chen H, Zhou H, Wang Z, Zhou H. Wenzi Jiedu Recipe ameliorates colorectal cancer by remodeling the gut microbiota and tumor microenvironment. Front Oncol. 2022;12: 915498. 36212428 10.3389/fonc.2022.915498 PMC9541612

[120] Wang Y, Zhang X, Li J, Zhang Y, Guo Y, Chang Q, et al. Sini decoction ameliorates colorectal cancer and modulates the composition of gut microbiota in mice. Front Pharmacol. 2021;12: 609992. 33776762 10.3389/fphar.2021.609992 PMC7991589

[121] Nie X, Geng Z, Liu J, Qi L, Wang Z, Liu T, et al. Chinese herbal medicine anticancer cocktail soup activates immune cells to kill colon cancer cells by regulating the gut microbiota-Th17 axis. Front Pharmacol. 2022;13: 963638. 36147322 10.3389/fphar.2022.963638 PMC9486099

[122] Vetizou M, Pitt JM, Daillere R, Lepage P, Waldschmitt N, Flament C, et al. Anticancer immunotherapy by CTLA-4 blockade relies on the gut microbiota. Science. 2015;350(6264):1079–84. 26541610 10.1126/science.aad1329 PMC4721659

[123] Sharpe AH, Pauken KE. The diverse functions of the PD1 inhibitory pathway. Nat Rev Immunol. 2018;18(3):153–67. 28990585 10.1038/nri.2017.108

[124] Tison A, Garaud S, Chiche L, Cornec D, Kostine M. Immune-checkpoint inhibitor use in patients with cancer and pre-existing autoimmune diseases. Nat Rev Rheumatol. 2022;18(11):641–56. 36198831 10.1038/s41584-022-00841-0

[125] Kornepati AVR, Vadlamudi RK, Curiel TJ. Programmed death ligand 1 signals in cancer cells. Nat Rev Cancer. 2022;22(3):174–89. 35031777 10.1038/s41568-021-00431-4 PMC9989967

[126] Wei SC, Duffy CR, Allison JP. Fundamental mechanisms of immune checkpoint blockade therapy. Cancer Discov. 2018;8(9):1069–86. 30115704 10.1158/2159-8290.CD-18-0367

[127] Wang M, Du Q, Jin J, Wei Y, Lu Y, Li Q. LAG3 and its emerging role in cancer immunotherapy. Clin Transl Med. 2021;11(3): e365. 33784013 10.1002/ctm2.365 PMC7989707

[128] Nayak L, Iwamoto FM, LaCasce A, Mukundan S, Roemer MGM, Chapuy B, et al. PD-1 blockade with nivolumab in relapsed/refractory primary central nervous system and testicular lymphoma. Blood. 2017;129(23):3071–3. 28356247 10.1182/blood-2017-01-764209 PMC5766844

[129] Le DT, Uram JN, Wang H, Bartlett BR, Kemberling H, Eyring AD, et al. PD-1 blockade in tumors with mismatch-repair deficiency. N Engl J Med. 2015;372(26):2509–20. 26028255 10.1056/NEJMoa1500596 PMC4481136

[130] Nusse R, Clevers H. Wnt/beta-catenin signaling, disease, and emerging therapeutic modalities. Cell. 2017;169(6):985–99. 28575679 10.1016/j.cell.2017.05.016

[131] Peluso I, Yarla NS, Ambra R, Pastore G, Perry G. MAPK signalling pathway in cancers: olive products as cancer preventive and therapeutic agents. Semin Cancer Biol. 2019;56:185–95. 28912082 10.1016/j.semcancer.2017.09.002

[132] Han Y, Zhang Y, Tian Y, Zhang M, Xiang C, Zhen Q, et al. The interaction of the IFNgamma/JAK/STAT1 and JAK/STAT3 signalling pathways in EGFR-mutated lung adenocarcinoma cells. J Oncol. 2022;2022:9016296. 36185620 10.1155/2022/9016296 PMC9519310

[133] Zhang Z, Richmond A, Yan C. Immunomodulatory Properties of PI3K/AKT/mTOR and MAPK/MEK/ERK Inhibition Augment Response to Immune Checkpoint Blockade in Melanoma and Triple-Negative Breast Cancer. Int J Mol Sci. 2022. 10.3390/ijms23137353. 35806358 10.3390/ijms23137353 PMC9266842

[134] Necchi A, Joseph RW, Loriot Y, Hoffman-Censits J, Perez-Gracia JL, Petrylak DP, et al. Atezolizumab in platinum-treated locally advanced or metastatic urothelial carcinoma: post-progression outcomes from the phase II IMvigor210 study. Ann Oncol. 2017;28(12):3044–50. 28950298 10.1093/annonc/mdx518 PMC5834063

[135] Lee YR, Chen M, Pandolfi PP. The functions and regulation of the PTEN tumour suppressor: new modes and prospects. Nat Rev Mol Cell Biol. 2018;19(9):547–62. 29858604 10.1038/s41580-018-0015-0

[136] Matozaki T, Murata Y, Okazawa H, Ohnishi H. Functions and molecular mechanisms of the CD47-SIRPalpha signalling pathway. Trends Cell Biol. 2009;19(2):72–80. 19144521 10.1016/j.tcb.2008.12.001

[137] Sun Y, Revach OY, Anderson S, Kessler EA, Wolfe CH, Jenney A, et al. Targeting TBK1 to overcome resistance to cancer immunotherapy. Nature. 2023;615(7950):158–67. 36634707 10.1038/s41586-023-05704-6 PMC10171827

[138] Abdel-Hafiz HA, Schafer JM, Chen X, Xiao T, Gauntner TD, Li Z, et al. Y chromosome loss in cancer drives growth by evasion of adaptive immunity. Nature. 2023;619(7970):624–31. 37344596 10.1038/s41586-023-06234-x PMC10975863

[139] Quezada SA, Peggs KS, Curran MA, Allison JP. CTLA4 blockade and GM-CSF combination immunotherapy alters the intratumor balance of effector and regulatory T cells. J Clin Invest. 2006;116(7):1935–45. 16778987 10.1172/JCI27745 PMC1479425

[140] Li X, Zhong J, Deng X, Guo X, Lu Y, Lin J, et al. Targeting myeloid-derived suppressor cells to enhance the antitumor efficacy of immune checkpoint blockade therapy. Front Immunol. 2021;12: 754196. 35003065 10.3389/fimmu.2021.754196 PMC8727744

[141] Kumari N, Choi SH. Tumor-associated macrophages in cancer: recent advancements in cancer nanoimmunotherapies. J Exp Clin Cancer Res. 2022;41(1):68. 35183252 10.1186/s13046-022-02272-x PMC8857848

[142] Zhang H, Yue X, Chen Z, Liu C, Wu W, Zhang N, et al. Define cancer-associated fibroblasts (CAFs) in the tumor microenvironment: new opportunities in cancer immunotherapy and advances in clinical trials. Mol Cancer. 2023;22(1):159. 37784082 10.1186/s12943-023-01860-5 PMC10544417

[143] Dubrot J, Du PP, Lane-Reticker SK, Kessler EA, Muscato AJ, Mehta A, et al. In vivo CRISPR screens reveal the landscape of immune evasion pathways across cancer. Nat Immunol. 2022;23(10):1495–506. 36151395 10.1038/s41590-022-01315-x

[144] Driesen J, Popov A, Schultze JL. CD25 as an immune regulatory molecule expressed on myeloid dendritic cells. Immunobiology. 2008;213(9–10):849–58. 18926299 10.1016/j.imbio.2008.07.026

[145] Yang Z, Xu G, Wang B, Liu Y, Zhang L, Jing T, et al. USP12 downregulation orchestrates a protumourigenic microenvironment and enhances lung tumour resistance to PD-1 blockade. Nat Commun. 2021;12(1):4852. 34381028 10.1038/s41467-021-25032-5 PMC8357983

[146] Ma S, Zhao Y, Lee WC, Ong LT, Lee PL, Jiang Z, et al. Hypoxia induces HIF1alpha-dependent epigenetic vulnerability in triple negative breast cancer to confer immune effector dysfunction and resistance to anti-PD-1 immunotherapy. Nat Commun. 2022;13(1):4118. 35840558 10.1038/s41467-022-31764-9 PMC9287350

[147] Barboy O, Bercovich A, Li H, Eyal-Lubling Y, Yalin A, Shapir Itai Y, et al. Modeling T cell temporal response to cancer immunotherapy rationalizes development of combinatorial treatment protocols. Nat Cancer. 2024. 10.1038/s43018-024-00734-z. 38429414 10.1038/s43018-024-00734-z

[148] Chow A, Perica K, Klebanoff CA, Wolchok JD. Clinical implications of T cell exhaustion for cancer immunotherapy. Nat Rev Clin Oncol. 2022;19(12):775–90. 36216928 10.1038/s41571-022-00689-z PMC10984554

[149] Wei F, Su Y, Quan Y, Li X, Zou Q, Zhang L, et al. Anticoagulants enhance molecular and cellular immunotherapy of cancer by improving tumor microcirculation structure and function and redistributing tumor infiltrates. Clin Cancer Res. 2023;29(13):2525–39. 36729148 10.1158/1078-0432.CCR-22-2757

[150] Zhou C, Wang Y, Zhao J, Chen G, Liu Z, Gu K, et al. Efficacy and biomarker analysis of Camrelizumab in combination with Apatinib in patients with advanced Nonsquamous NSCLC previously treated with chemotherapy. Clin Cancer Res. 2021;27(5):1296–304. 33323401 10.1158/1078-0432.CCR-20-3136

[151] Qian C, Zhou Y, Zhang T, Dong G, Mengyao Song Y, Tang ZW, Suyun Y, Shen Q, Chen W, Choi JP, Yan J, Zhong C, Wan L, Li J, Wang A, Yin L, Zhao Y. Targeting PKM2 signaling cascade with salvianic acid A normalizes tumor blood vessels to facilitate chemotherapeutic drug delivery. Acta Pharmaceutica Sinica B. 2024. 10.1016/j.apsb.2024.02.003. 38799619 10.1016/j.apsb.2024.02.003 PMC11121179

[152] Zhao SY, Yin SS, Wang R, Yu HY. Application of promoting blood circulation and removing blood stasis drugs in anti-malignant tumor metastasis. Tianjin Univ Tradit Chin Med. 2020;39(2):231–6.

[153] Li J, Wu C, Hu H, Qin G, Wu X, Bai F, et al. Remodeling of the immune and stromal cell compartment by PD-1 blockade in mismatch repair-deficient colorectal cancer. Cancer Cell. 2023;41(6):1152-69 e7. 37172580 10.1016/j.ccell.2023.04.011

[154] Sun X, Wu B, Chiang HC, Deng H, Zhang X, Xiong W, et al. Tumour DDR1 promotes collagen fibre alignment to instigate immune exclusion. Nature. 2021;599(7886):673–8. 34732895 10.1038/s41586-021-04057-2 PMC8839149

[155] Felip E, Altorki N, Zhou C, Vallieres E, Martinez-Marti A, Rittmeyer A, et al. Overall survival with adjuvant atezolizumab after chemotherapy in resected stage II-IIIA non-small-cell lung cancer (IMpower010): a randomised, multicentre, open-label, phase III trial. Ann Oncol. 2023;34(10):907–19. 37467930 10.1016/j.annonc.2023.07.001

[156] Zhao J, Wang L, Zhou A, Wen S, Fang W, Zhang L, et al. Decision model for durable clinical benefit from front- or late-line immunotherapy alone or with chemotherapy in non-small cell lung cancer. Med. 2024;5(8):981–974. 38781965 10.1016/j.medj.2024.04.011

[157] Chen J, Larsson L, Swarbrick A, Lundeberg J. Spatial landscapes of cancers: insights and opportunities. Nat Rev Clin Oncol. 2024;21(9):660–74. 39043872 10.1038/s41571-024-00926-7

[158] Lee S, Kim G, Lee J, Lee AC, Kwon S. Mapping cancer biology in space: applications and perspectives on spatial omics for oncology. Mol Cancer. 2024;23(1):26. 38291400 10.1186/s12943-024-01941-z PMC10826015

[159] Guo C, Kong L, Xiao L, Liu K, Cui H, Xin Q, et al. The impact of the gut microbiome on tumor immunotherapy: from mechanism to application strategies. Cell Biosci. 2023;13(1):188. 37828613 10.1186/s13578-023-01135-y PMC10571290

